# Azithromycin vs. Placebo for the Clinical Outcome in *Campylobacter concisus* Diarrhoea in Adults: A Randomized, Double-Blinded, Placebo-Controlled Clinical Trial

**DOI:** 10.1371/journal.pone.0166395

**Published:** 2016-11-28

**Authors:** Hans Linde Nielsen, Karina Frahm Kirk, Jacob Bodilsen, Tove Ejlertsen, Henrik Nielsen

**Affiliations:** 1 Department of Infectious Diseases, Aalborg University Hospital, Aalborg, Denmark; 2 Department of Clinical Microbiology, Aalborg University Hospital, Aalborg, Denmark; 3 Department of Clinical Medicine, Aalborg University, Aalborg, Denmark; Hvidovre Hospital, DENMARK

## Abstract

*Campylobacter concisus* has been associated with prolonged mild diarrhoea, but investigations regarding the efficacy of antimicrobial treatment have not been reported previously. We initiated a phase 3, single-centre, randomized, double-blinded, placebo-controlled study comparing the efficacy of 500 mg once-daily dose of azithromycin with a 500 mg once-daily dose of placebo for three days, for the treatment of *C*. *concisus* diarrhoea in adult patients with a follow-up period of ten days. If symptoms persisted at day ten, the patient was offered cross-over study treatment of three days and another ten-day follow-up period. The primary efficacy endpoint was the clinical response, defined as time to cessation of diarrhoea (<3 stools/day or reversal of accompanying symptoms). Our estimated sample size was 100 patients. We investigated a total of 10,036 diarrheic stool samples from 7,089 adult patients. Five-hundred and eighty-eight *C*. *concisus* positive patients were assessed for eligibility, of which 559 were excluded prior to randomization. The three main reasons for exclusion were duration of diarrhoea longer than 21 days (n = 124), previous antibiotic treatment (n = 113), and co-pathogens in stools (n = 87). Therefore, 24 patients completed the trial with either azithromycin (n = 12) or placebo (n = 12). Both groups presented symptoms of mild, prolonged diarrhoea with a mean duration of 18 days (95% CI: 16–19). One person in the azithromycin group and four from the placebo group chose to continue with crossover medication after the initial ten-day period. In the azithromycin group, there was a mean of seven days (95% CI: 5–9) to clinical cure and for the placebo group it was ten days (95% CI: 6–14) (OR—3 (95% CI: -7–1). We observed no differences in all examined outcomes between azithromycin treatment and placebo. However, due to unforeseen recruitment difficulties we did not reach our estimated sample size of 100 patients and statistical power to conclude on an effect of azithromycin treatment was not obtained.

***Trial Registration*:** Clinicaltrials.gov identifier: NCT01531218.

## Introduction

*Campylobacter jejuni* is the most common bacterial cause of human diarrhoeal disease [1]. The infection is usually self-limited and antimicrobial therapy reduces the duration of intestinal symptoms by approximately one day only, when compared to placebo [2]. Consequently, antimicrobial therapy is warranted only for high-risk patients with severe *C*. *jejuni* infection, and a macrolide antibiotic (e.g. azithromycin) is generally recommended as the first drug of choice [3]. In recent years, emerging evidence has brought attention to the potential clinical significance of a related species, *Campylobacter concisus*, originally isolated from patients with periodontal lesions and now considered a part of the normal human oral microbiota [4, 5].

*Campylobacter concisus* has shown abilities of epithelial adherence and invasion in in-vitro cell-lines [6]. A study of HT-29/B6 cells exposed to *C*. *concisus* showed increased production of lactate-dehydrogenase as well as induction of apoptotic leaks and tight junction alterations that may lead to an impaired barrier function, demonstrating a leak-flux mechanism that parallels the clinical manifestation of diarrhoea [7]. Furthermore, in a recent population based study from Denmark *C*. *concisus* was more prevalent than *C*. *jejuni* in diarrheic stool samples [8].

In two questionnaire studies, adult patients and parents of children infected with *C*. *concisus* reported a milder, though more prolonged diarrhoea compared to patients infected with *C*. *jejuni* [9, 10]. Half of the adult *C*. *jejuni* patients reported receiving antibiotic treatment prescribed by their general practitioner (GP), whereas one-third of *C*. *concisus-*positive patients reported treatment with either oral ciprofloxacin or a macrolide [9]. Investigations into the efficacy of antimicrobial treatment of diarrhoea due to *C*. *concisus* infection have not previously been performed. Therefore, we initiated a study comparing the efficacy and safety of azithromycin with placebo for 3 days for the treatment of diarrhoea in *C*. *concisus* positive adult patients.

## Material and Methods

### Study design

This was an investigator initiated, phase 3, single-centre, randomized, double-blinded, placebo-controlled study comparing the efficacy and safety of 500 mg once-daily dose of azithromycin with a 500 mg once-daily dose of placebo for 3 days for the treatment of diarrhoea in *C*. *concisus* positive adult patients (NCT01531218, https://clinicaltrials.gov). Participants were asked to self-report diarrheic symptoms for a follow-up period of initially ten days. If the patient had ongoing diarrheic symptoms (i.e. no effect of either azithromycin or placebo) after the initial ten-day observational period, the patient was offered cross-over treatment, also double-blinded, succeeded by another ten-day follow up period. (Fig 2). The study was conducted in the North Denmark Region, with a population of 578,839 inhabitants.

### Bacteriology

Diarrheic stool samples were cultured at the Department of Clinical Microbiology, Aalborg University Hospital combining a previously described filter method with routine diagnostic methods. [11]. *Campylobacter concisus* was isolated by the filter technique on 5% horse blood agar plates, containing 1% yeast extract (SSI Diagnostica, Hillerød, Denmark) and incubated at 37°C in a microaerobic atmosphere with 3% hydrogen. Final identification was confirmed by MALDI-TOF analysis (BRUKER DALTONIK GmbH, Bremen, Germany) as well as by species-specific real-time PCR based on the *cpn60* gene, as described elsewhere [12,13]. Antimicrobial susceptibility testing to azithromycin was performed with use of disk diffusion with 15μg azithromycin using EUCAST-and CLSI potency NEO-SENSITABS™ (Rosco Diagnostica A/S, Taastrup, Denmark) according to the manufacturer’s instructions. Stools from patients eligible for inclusion in the study, were also examined by PCR-based methods for diarrheic *Escherichia coli*, norovirus, rotavirus, adenovirus, and the protozoes *Giardia lamblia*, *Entamoeba histolytica* and *Cryptosporidium parvum*. Saliva samples were also cultivated by use of the filter method as described above.

### Subjects

#### Inclusion criteria

Study subjects were diarrheic adults ≥18 years, with a *C*. *concisus* culture-positive stool sample. Diarrhoea was defined as three or more loose stools per day, or two loose stools a day combined with at least one or more accompanying symptoms such as abdominal pain, nausea, vomiting or fever, persisting for at least 24 hours.

#### Exclusion criteria

Patients were excluded if they presented with diarrheic symptoms for more than 21 days, hypersensitivity to azithromycin or macrolides in general and if pregnant or lactating. They were also excluded if their stool sample was positive for one or more of the following pathogenic enteric bacteria: *Salmonella*, *Shigella*, *Yersinia*, *Campylobacter jejuni/coli*, *Aeromonas*, *Plesiomonas*, *Vibrio*, *Clostridium difficile*, or diarrheic *Escherichia coli*, as well as norovirus, rotavirus and adenovirus, and the protozoes *Giardia lamblia*, *Entamoeba histolytica* or *Cryptosporidium parvum*. Antibiotic therapy within the last 4 weeks, colostomy or ileostomy, severe renal or liver disease, congenital or acquired QT-prolongation or other heart conditions, as well as inflammatory bowel disease (IBD), microscopic colitis or other chronic diarrheal disease were other exclusion criteria. Finally, patients were excluded if they were unable to follow the study protocol (e.g. due to dementia).

### Randomization, Double-blinding and Treatment

Patients meeting eligibility criteria were randomly assigned in a one to one ratio to receive either azithromycin or placebo (Lactose Monohydrate) supplied as identical 500 mg capsules approved by the Danish Health and Medicines Authority. A Pharmacist (http://www.glostrup-apotek.dk/) consecutively listed randomisation for patients 1–100. The list was blinded for the Investigator and treatment allocation remained blinded until trial completion. However, in case of acute need for breach of code, the Pharmacist provided round the clock service Azithromycin and matching placebo were taken once daily for three days.

### Assessment

The primary efficacy endpoint was clinical response to the azithromycin and placebo regimens, defined as time to cessation of diarrhoea (<3 stools/day or reversal of accompanying symptoms). When a stool sample tested positive for *C*. *concisus*, the patient was contacted by telephone by their GP or responsible physician if hospitalized. In a primary interview with the investigator, subjects were asked about their general health, previous medical records, and diarrheic symptoms. At first visit (day 1) the patient met the principal-investigator in an outpatient setting and was asked about the following general symptoms: headache, fatigue, sleeping difficulties, dizziness, itching, sensory disturbances, joint pain, back pain, dyspnoea, angina pectoris, and sweating. Hereafter, the patient was randomized to either azithromycin or placebo 500 mg once-daily dose for 3 days for the treatment of diarrhoea and blood (C-reactive Protein (CRP) and leukocytes) and saliva samples were collected. Subsequently, the participants answered daily questionnaires regarding diarrheic symptoms, number of stools per day, clinical symptoms and possible side effects to the medication for as long as symptoms persisted, or a total follow-up period of ten days. On day ten, the patient was contacted by telephone by the principal-investigator and asked about their current status. If patients stated ongoing diarrheic symptoms (i.e. no effect of either azithromycin or placebo) the patient was offered cross-over study treatment (azithromycin or placebo), also double-blinded, for three days, followed by another ten-day follow-up period. A secondary bacteriological endpoint was eradication of *C*. *concisus* in stool and saliva ten days after primary enrolment, defined as culture negative stool and saliva samples on day ten, using the methods described above.

### Sample Size Calculation and Statistical Analysis

Statistical analysis was conducted using Stata® software, version 10 (College Station, Texas). The sample size was calculated to achieve appropriate statistical power (β = 0.80), with the acceptable type 1 error (α = 0.05), type 2 error (b = 0.20) and hypothesis that the OR for cure in the azithromycin treatment arm versus placebo was at least three. The required sample with the aforementioned assumptions was 44 subjects per treatment arm. With an expected 10% withdrawal of subjects during the trial, the final sample size was estimated to 50 subjects in each group. Continuous variables were compared using the student t-test, whereas categorical variables were compared using Fisher’s exact test with the statistical significance level set to 0.05 Estimates for clinical efficacy were calculated by linear regression for parametric values (‘regress’ procedure in Stata) and rounded up to the nearest integer (days). Binary values were analysed by logistic regression (‘logit’ procedure in Stata).

## Results

### Demographics and patient inclusion

**Fig 1:**
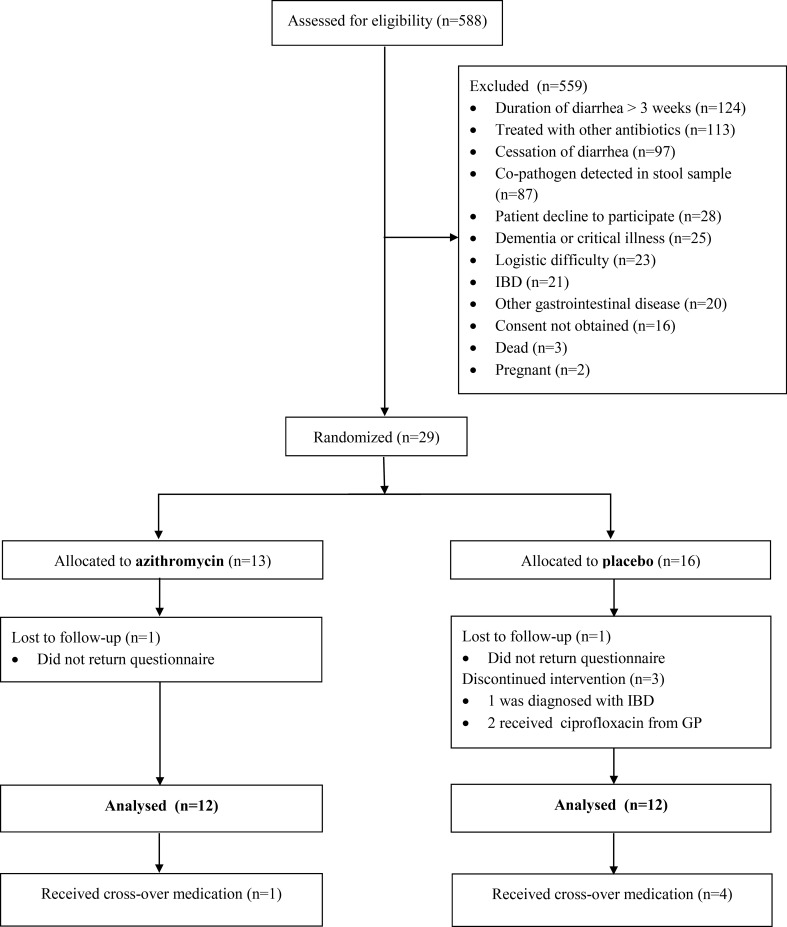
Flow-chart of subject inclusion.

The study was performed from 1. April 2012 till 30. November 2013 and based on routine diarrheic stool samples sent to the Department of Clinical Microbiology at Aalborg University Hospital for cultivation of enteric pathogenic bacteria, from both General Practitioners and Hospital Wards. During the study period, we investigated a total of 10,036 diarrheic stool samples from 7,089 adult patients with a male to female ratio of 3065/4024 and with a mean age of 54 years (95%CI: 53–54). Five hundred and eighty-eight unique adult patients with a male to female ratio of 254/334 and with a mean age of 55 years (95% CI: 53–56) had *C*. *concisus* in stools and were assessed for eligibility, but prior to randomization, 559 had to be excluded according to study in- and exclusion criteria (Fig 1). The main reasons for exclusion were duration of diarrhoea for more than 3 weeks (n = 124) and antibiotic treatment prior to inclusion (n = 113). Co-pathogens were detected in 87 subject’s stools; the majority of these were *Clostridium difficile*. Twenty-one were excluded due to a preceding IBD diagnosis and 20 patients had other gastrointestinal diseases, primarily being microscopic colitis. Due to the high exclusion rate, we did not reach our estimated sample size of 100 patients. In total, the intention-to-treat group comprised of 29 subjects that were randomized to treatment. Two subjects did not return questionnaires and were lost to follow-up and one patient was diagnosed with IBD shortly after inclusion. Two patients did not improve during the study treatment and were prescribed ciprofloxacin for continued diarrheic symptoms by their GP. Baseline characteristics of the intention to treat population was compared to the per protocol population and there was no difference between the two groups (data not shown). The per-protocol population that completed the trial was twenty-four subjects in total, of which five (one azithromycin, four placebo) chose to continue with cross-over treatment after the initial 10-day period (Fig 1).

**Fig 2:**
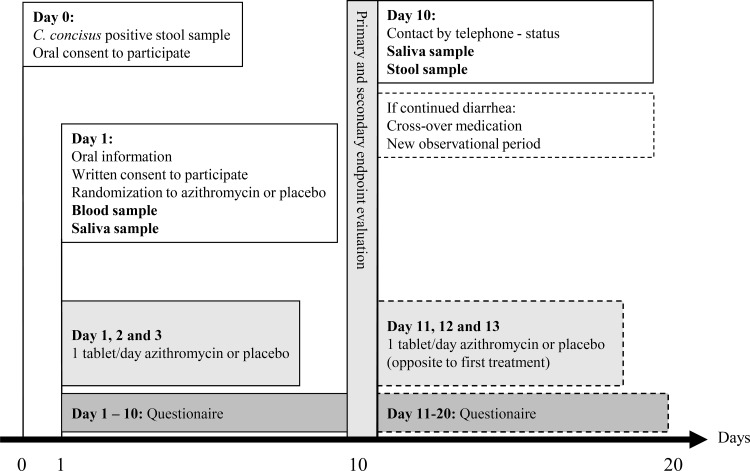
Upon diagnosis of *C*. *concisus* in a stool sample the diarrheic patient was contacted by telephone, through their General Practitioner or physician (if hospitalized), by the principal-investigator (day 0). If the patient still had diarrhoea the patient was offered to participate in the clinical trial. At day 1 the patient met the principal-investigator in an outpatient setting and was randomized to either azithromycin or placebo 500 mg once-daily dose for three days for the treatment of diarrhoea. Moreover, the patient was asked to deliver a blood sample (CRP and leukocytes) as well as a saliva sample. During the follow-up period of ten days the patient had to fill in a questionnaire on daily basis regarding diarrheic symptoms. At day ten the patient was contacted by telephone by the principal-investigator and if the patient stated ongoing diarrheic symptoms (no effect of study treatment; azithromycin or placebo) the patient was offered another ten-day study period and treatment with cross-over study medication (azithromycin or placebo), also double-blinded. Finally, the patient was asked to deliver a saliva sample as well as a stool sample for an ongoing diagnosis of *C*. *concisus* infection.

### Patient characteristics

Examining all 24 patients in the per-protocol population revealed a mean age of 44 years (95% CI: 37–52) and a male to female ratio of 9:15. Baseline demographics were similar in both treatment groups (n = 12 in both groups) with a trend toward younger age in the placebo group (p = 0.055) (Table 1). Half of the patients had pre-existing comorbidities including arterial hypertension, depression, migraine and osteoporosis, but none of these were associated with gastrointestinal illness (data not shown). Whilst 6/24 (25%) reported daily fatigue, no other general symptom was reported. Baseline characteristics and clinical presentations of the intention-to-treat population did not differ from the per protocol populations (data not shown).

**Table 1:** Baseline characteristics and diarrheic symptoms at inclusion for per-protocol population allocated to either azithromycin or placebo. For continuous variables, 95% confidence intervals are presented in parentheses following mean values.

Variable	Azithromycin (n = 12)	Placebo (n = 12)	p-value
Sample origin (GP/hospital ward)	11/1	11/1	1.00
Sex (male/female)	6/6	3/9	0.20
Age, mean years	50 (39.5–61)	38 (27–50)	0.055
BMI (kg/m^2^) (mean)	23.9 (21.2–26.6)	24.2 (21.6–27.0)	0.58
C-reactive protein (mg/L) (mean)	7.6 (0.0–16)	4.9 (1.12–8.71)	0.27
Leucocytes (10^9^/L) (mean)	6.9 (5.96–7.9)	6.48 (5.31–7.6)	0.25
Duration of diarrhoea, days (mean)	19 (17–20)	17 (14–19)	0.10
Nocturnal diarrhoea (yes/no)	9/3	8/4	1.00
Max. stools/day (mean)	10 (7–13)	11 (6–15)	0.62
Consistency of stools (yes/no)			
Watery	11/1	12/0	1.00
Mucus in stool	6/6	7/5	1.00
Blood in stool	1/11	0/12	1.00
Symptoms (yes/no)			
Fever	2/10	4/8	0.53
Chills	2/10	4/8	0.53
Nausea	4/8	7/5	0.41
Vomiting	1/11	3/9	0.59
Headache	2/10	8/4	0.04
Dizziness	4/8	1/11	0.32
Abdominal pain	8/4	9/3	1.00
Muscle aches	2/10	2/10	1.00
Weight loss (yes/no)	8/4	6/6	0.68

### Clinical presentation

The clinical presentation of the study subjects clearly reflected the symptoms previously reported in gastroenteritis due to *C*. *concisus* [9], and were similar in the two groups (Table 1). The overall duration of diarrhoea at the time of inclusion was a median of 18 days (95% CI: 16–19). Seventeen of the 24 (71%) subjects reported nocturnal diarrhoea and half of the patients had mucus in stool, while only one patient reported bloody stools. One-fourth reported having had a fever (temperature >37,5°C) and chills at some time during the course of illness, while only 2 patients (8%) had fever at the time of inclusion. Headache was reported by eight patients in the placebo group, and two in the azithromycin group (p = 0.04). Overall, 17 (71%) patients reported abdominal pain and 14 (58%) reported weight loss, with a fairly even distribution between the two groups. Finally, only four patients (two in each group) had elevated CRP (>10.0 mg/L) at inclusion, whereas all other patients had normal blood parameters.

### Primary outcome

The primary outcome was time to cure, defined as time to cessation of diarrhoea (<3 stools/day or reversal of accompanying symptoms). For the azithromycin group 9/12 (75%) subjects reported cessation of diarrhoea after the course of antibiotics. For the placebo group, it was 7/12 (58%) (OR 2.14 (95% CI: 0.38–12.2). One person in the azithromycin group and four from the placebo group chose to continue with crossover medication after the initial 10-day period. In the azithromycin group, there was a mean seven days (95% CI: 5–9) to clinical cure, and for the placebo group it was ten days (95% CI: 6–14) (OR—3 (95% CI: -7–1).

Patients were asked to report symptoms on a daily basis, analogous to the questions at inclusion. On day five after treatment allocation, one person in the azithromycin group reported continued headache, and three people abdominal pain. In the placebo group, two people reported nausea, three reported headaches, three suffered from dizziness and seven from abdominal pain. On day 10, no participant from the azithromycin group reported any symptom other than diarrhoea. In the placebo group, two still reported nausea, two complained of headaches, one still suffered from dizziness and five from abdominal pain. We have limited statistical power to show a significant difference between the two groups, but it seems that there was a trend towards faster recovery of symptoms in the azithromycin treatment group, compared to the placebo group.

### Secondary outcome

All patients, in both the azithromycin and placebo group, had a negative *C*. *concisus* stool test ten days after inclusion. Similarly, while 5/12 subjects in both groups had *C*. *concisus* in their saliva at inclusion, and all but one subject in the placebo group tested negative on day 10.

### Treatment compliance

Subjects were required to document their intake of the study medication on each day in the questionnaires provided, and asked to bring unused medication, if any, to follow-up visit on day ten. No subjects in the trial reported compliance failure.

### Safety

No suspected unexpected serious adverse reactions (SUSAR) or treatment related severe adverse events (SAE) were reported, and no subjects discontinued the study due to adverse effects.

## Discussion

This is the first study to assess the clinical efficacy of antibiotic treatment of *C*. *concisus* associated diarrhoea. However, due to unforeseen difficulty with recruitment, the sample size does not have statistical power to finally conclude whether patients should be treated with azithromycin or not, leaving the main question unanswered. While there was a trend toward shorter disease duration with azithromycin treatment, the sample size is too small for the results to be conclusive. On the other hand, the study confirms our previous studies that *C*. *concisus* is often isolated from human diarrheic stool samples [8, 14], and may lead to prolonged, mild diarrhoea [9, 10].

One-sixth of our previous isolates of *C*. *concisus* were resistant to ciprofloxacin, whereas we recorded almost no resistance to azithromycin [14]. The isolates in the present study were all susceptible to macrolides, and azithromycin is generally recommend as the drug of choice if antibiotic treatment is indicated for severe infection due to *C*. *jejuni*. However, antibiotic treatment of acute gastroenteritis is usually not recommended in adults without comorbidity [3]. Nevertheless, one-fifth of subjects eligible for enrolment in this study had already been treated with antibiotics by their primary physician, possibly because they presented with more severe diarrhoae. However, it remains unknown whether these patients had any effect of the antimicrobial therapy prescribed, and we were not able to conduct a prospective follow-up, since they were in the out-patient setting. During the study period, two patients in the placebo group were prescribed ciprofloxacin by their GP due to persistent symptoms, and had to be excluded. This is a reflection of the nature of diarrheic infection due to *C*. *concisus*, and a major limitation when assessing treatment options, as patients already reported diarrhoea duration for a median of 19 days before enrolment.

In the initiation of the trial, we limited inclusion to patients with duration of diarrhoea for a maximum of three weeks only. However, it is well known that *C*. *concisus* infection is associated to prolonged diarrhoea and 21% of eligible participants reported duration of diarrhoea longer than three weeks and had to be excluded. This also reflects the clinical course of *C*. *concisus* associated diarrhoea as previously described [9, 10], where patients are symptomatic for weeks, but not affected by general malaise and fever, as seen in *C*. *jejuni* infections. Patients will therefore usually only consult their physician late in the course of illness.

Studies have found a high prevalence of *C*. *concisus* DNA in mucosal biopsies from patients with inflammatory bowel disease (IBD) [15–18], and we previously reported that 12% of patients with *C*. *concisus* associated diarrhoea were diagnosed with microscopic colitis six months after infection. [9]. Consequently, if limitations in the duration of diarrheic symptoms were not included in this study, there could be a potential risk of undiagnosed IBD or microscopic colitis cases. This was exemplified by a subject in the placebo group who was diagnosed with IBD (Crohn's Disease) a few days after inclusion, and therefore consequently excluded.

In the small group of subjects to complete this trial, there was no statistically significant difference in clinical or bacteriological efficacy between the two groups. Not surprisingly, participants presented with a mean duration of diarrhoea of 19 days. Half of the patients had mucus in stools and more than two-thirds had nocturnal diarrhoea, also in accordance with previously findings [9]. Even though half of the patients reported mucus in their stool, the *C*. *concisus* infection still appeared localized in the gut, exerting no major systemic response—only one-fourth reported having had a fever and all but four patients presented with normal CRP levels at inclusion. At baseline there was no overall difference between the two small groups, although headache was more prevalent in the placebo group, which also had more females (not significant).

After randomization there was a trend towards faster recovery of symptoms in the azithromycin group compared to the placebo group and while this should interpreted with caution, it could mimic the effects of antibiotic treatment of *C*. *jejuni* infections that may shorten the duration of clinical symptoms only slightly [3]. Only one patient in the azithromycin group requested crossover treatment, whereas four patients in the placebo group requested azithromycin after the initial ten-day study period. The patients in the azithromycin group had a two-day shorter time to clinical cure compared to the patients in the placebo group, but this may merely reflect the chronic nature of *C*. *concisus* diarrhoea.

Before the study, we had no data describing the duration of *C*. *concisus* excretion in stool. Interestingly, all patients had a negative *C*. *concisus* stool test ten days after inclusion independently of treatment with azithromycin or placebo, suggesting that infection was cleared by immune cells, rather than antibiotics. There was also a difference in the mean age between the azithromycin and placebo groups (50 vs. 38). Since the infection could be cleared by immune cells rather than antibiotics and the efficiency of the immune system declines with age, age may also have acted as a confounding variable.

A similar trial, conducted earlier in the course of illness would be of great significance in elucidating the effects of azithromycin treatment, but the natural course of *C*. *concisus* diarrhoea limits this possibility. Moreover, Danish Ethical regulations prohibit the enrolment of patients from general practice by others than the patient’s own GP, impeding recruitment time for the majority of patients.

We cannot exclude selection bias in the decision for microbiological examination, as stool samples were requested by patients’ GP’s or hospital doctors. However, we included only culture-positive cases of *C*. *concisus*, and excluded stool samples with other identified gastroenterological pathogens. Fifteen percent of *C*. *concisus* positive patients also had a co-pathogen detected in stools, the majority of these being *Clostridium difficile*, in agreement with our previous findings [14].

In conclusion, this is the first study to assess clinical efficacy of antibiotic treatment of *C*. *concisus* associated diarrhoea. The study confirms our previous findings, that *C*. *concisus* is often isolated from human diarrheic stool samples, and may lead to prolonged, mild diarrhoea. We observed a trend towards shorter disease duration with azithromycin treatment and faster symptom recovery.

However, due to unforeseen recruitment difficulties we did not reach our estimated sample size of 100 patients and statistical power to conclude on an effect of azithromycin treatment was not obtained.

## Supporting Information

S1 File Protocol (Danish). (PDF)

S2 File Protocol (English). (PDF)

S3 File Patient data. (XLSX)

S4 File CONSORT 2010 Checklist. (PDF)

S5 File Questionnaire day 1–10 (Danish). (PDF)

S6 File Questionnaire day 1–10 (English). (PDF)

## Abbreviations

IQR: Inter quartile range
GP: General practitioner
IBD: Inflammatory bowel disease
CRP: C-reactive Protein
OR: Odds ratio
SUSAR: Suspected unexpected serious adverse reactions
SAE: severe adverse events
