# Supplementary material for: Azithromycin vs. Placebo for the Clinical Outcome in Campylobacter concisus Diarrhoea in Adults: A Randomized, Double-Blinded, Placebo-Controlled Clinical Trial
Source: PLoS One. 2016 Nov 28;11(11):e0166395. doi: 10.1371/journal.pone.0166395 (PMC5125586; doi:10.1371/journal.pone.0166395)
Supplement: S1 File — (PDF) [file pone.0166395.s001.pdf]

Campylobacter concisus og bakteriel diarré

Et randomiseret, dobbelt blindet, placebo kontrolleret, parallel gruppe klinisk studie af azithromycin hos patienter med *Campylobacter concisus* og diarré

Sponsorinvestigator:

Hans Linde Nielsen, læge, ph.d.
Infektionsmedicinsk afdeling,
Aalborg Universitetshospital
Mølleparkvej 4, Postboks 365
9000 Aalborg
Telefon: 99 32 65 32
e-mail: halin@rn.dk

Sub-investigator:

Henrik Nielsen, Professor, Ledende Overlæge, dr. med.
Infektionsmedicinsk afdeling,
Aalborg Universitetshospital
Mølleparkvej 4, Postboks 365
9000 Aalborg
Telefon: 99 32 65 10
e-mail: Henrik.nielsen@rn.dk

Karina Frahm Kirk, Reservelæge
Infektionsmedicinsk afdeling
Aalborg Universitetshospital
Mølleparkvej 4, Postboks 365
9000 Aalborg
e-mail: jacob.bodilsen@rn.dk

Jacob Bodilsen, Kursusreservelæge
Infektionsmedicinsk afdeling
Aalborg Universitetshospital
Mølleparkvej 4, Postboks 365
9000 Aalborg
Telefon: 99 32 65 35
e-mail: jacob.bodilsen@rn.dk

Monitor:

GCP-enheden ved Aarhus Universitets Hospital
Olof Palmes Allé 15
8200 Aarhus N
Telefon: 99 32 62 44

Øvrige samarbejdspartnere og Videnskabelige medarbejdere:

Marie Nørgaard, kvalitetskoordinator
Klinisk Biokemisk Afd.
Aalborg Universitetshospital
Hobrovej 18-22
9000 Aalborg

Tove Ejlertsen, Overlæge, ph.d.
Klinisk Mikrobiologisk Afd.
Aalborg Universitetshospital
Mølleparkvej 8 A
9000 Aalborg
Telefon: 99 32 32 07
e-mail: Tove.ejlertsen@rn.dk

Glostrup apotek v. apoteker Steen Mejlgaard
Hovedvejen 101
2600 Glostrup
Telefon: 43 96 00 20
Fax: 43 96 07 67
e-mail: glostrup@apotekeren.dk
www.glostrup-apotek.dk

Baggrund

Bakteriel diarré

I befolkningen er der en høj forekomst af diarré sygdom, primært erhvervet igennem indtagelse af fødevarer, og *Campylobacter jejuni* er den hyppigste årsag til bakteriel diarré i hele den vestlige verden [1,2]. Familien af *Campylobacteraceae* omfatter i dag 21 species (*species*)., som er isoleret fra mennesker (www.bacterio.cict.fr). De udgør bl.a. *C. jejuni, C. coli, C. concisus, C. upsaliensis, C. lari, C. curvus, C. rectus, C. showae, C. gracilis, C. hominis, C. sputorum, C. fetus, C. hyointestinalis og C. lanienae.*
C. jejuni skønnes at være den hyppigste årsag til *Campylobacter* enteritis. *C. jejuni* findes som en del af den normale tarmflora hos en lang række af dyr, primært hos fugle. Smitten sker oftest via forurenede fødevarer, langt hyppigst i den industrielle forarbejdning af fjerkræ [3]. Diagnosen stilles ved dyrkning af bakterierne fra fæces og i alvorlige tilfælde ved dyrkning af bakterierne fra blodet. Afføringsprøver fra almen praksis og hospitalsafdelinger indsendt til kliniske mikrobiologiske afdelinger undersøges i rutinen kun for *C. jejuni* og *C. coli* [4]. Fordelingen mellem *C. jejuni* og *C. coli* skønnes til henholdsvis ca. 95 % og 5 %, og klinisk skelnes ikke mellem *C. jejuni* og *C. coli* [5]. I efterfølgende er de (*C. jejuni* og *C. coli*) kun nævnt som *C. jejuni.*

Forekomst af C. jejuni

I 2009 blev der i Danmark registreret 3352 tilfælde af *C. jejuni* infektioner [6]. Antallet af registrerede tilfælde udgør dog kun toppen af isbjerget, og det antages at langt flere personer med bakteriel diarré, har haft så milde sygdomsmanifestationer, at de ikke har søgt lægehjælp. Klinisk Mikrobiologisk afd. Aalborg Sygehus Syd udfører bakteriologiske undersøgelser for Region Nordjylland. I 2009 udførte afdelingen 16.035 fæcesdyrkninger fra 5.947 patienter og fandt 337 patienter med *C. jejuni.* Der anbefales 3 på hinanden følgende fæcesdyrkninger, for at opnå en større mulighed for et positivt fund [7]. Lokale data, fra Klinisk Mikrobiologisk afd. Aalborg Sygehus viser, at det kun er i ca. 12 % af alle bakterielle fæcesdyrkninger, at der kan dyrkes en bakteriologisk årsag til diarréen. De resterende 88 % kan have mange årsager, infektiøse som virus, parasitter og ikke identificerbare bakterier, men også mange non-infektiøse årsager som inflammatorisk tarmsygdom, bivirkninger til medicin, divertikulose og malabsorptionsygdomme mm. Enteritis med *C. jejuni* er hyppigst blandt børn og unge voksne, men kan forekomme alle aldre [8].

Sygdomsbillede ved *C. jejuni*

C. jejuni koloniserer først tyndtarmen, senere tyktarmen, og invaderer tarmslimhinden resulterende i en dysfunktion i tarmslimhinden, der giver diarré. Bakterien kan i sjældne tilfælde gå over i blodbanen og give en invasiv infektion [9]. Efter en inkubationsperiode på 1-3 døgn begynder sygdommen ofte med almen utilpashed, feber og influenzalignende symptomer. Derefter udvikles diarré, evt. blodig, ledsaget af kolikagtige mavesmerter. Sygdommen er næsten altid selvlimiterende og strækker sig fra få dage, og op til 1-2 uger [10]. Enteritis med *C. jejuni* præsenterer således et spektrum fra milde tilfælde til alvorlige sygdomsforløb, der kan nødvendiggøre indlæggelse. Behandlingen er væsketerapi og antibiotika efter resistensbestemmelse, oftest med makrolid-antibiotikum [11]. Efter en infektion med *C. jejuni* er det velkendt, at mellem 10 og 30 % af patienterne oplever vedvarende gener fra tarmgebetet i form af irriteret tyktarm [12-15]. Denne sygdom er karakteriseret ved ændrede afføringsvaner og intermitterende mavesmerter igennem mindst 3 måneder uden, at der kan påvises nogen organisk læsion jf. de såkaldte Rom kriterier [16]. Infektion med *C. jejuni* kan også give ekstraintestinale manifestationer i uger eller måneder efter den primære diarré episode, f.eks. i form af reaktiv artritis,

som ses i ca. 10 – 15 % af sygdomstilfældene [17]. Guillain-Barrés polyneuropati er en sjælden, men alvorlig komplikation. Sygdommen er oftest forudgået af en infektion, og *C. jejuni* blev for første gang i 1982 associeret til denne sygdom, og regnes i dag for den hyppiste udløsende årsag til sygdommen [18,19].

Forekomst af *C. concisus*

For andre *Campylobacter* species end *C. jejuni* og *C.coli*, er hyppigheden og evnen til at fremkalde diarré og evt. senkomplikationer ikke er så velbelyst. Ved den almindelige rutinedyrkning anvendes et selektivt dyrkningsmedium til *C. jejuni*, men det er velkendt at man med denne metode ikke kan dyrke andre *Campylobacter* species deriblandt *C. concisus*. Her skal afføringsprøven dyrkes vha. den såkaldte filtermetode i en mikroaerofil gasblanding, tilsat hydrogen [20]. Til endelig identifikation af et muligt *C. concisus* isolat anvendes molekylærdiagnostik vha. en species specific real-time-PCR baseret på *cpn60* genet [21].

En høj incidens af *C. concisus* blandt børn blev allerede beskrevet tilbage i 1995 af Lindblom et. al [22]. I et stort sydafrikansk studie, med brug af filtermetoden som en del af rutinedyrkningen på et børnehospital i Cape Town, fandt man over en 10-årig periode: 4.122 positve prøver med *Campylobacter* species ud af 19.535 afføringsprøver, og *C. concisus* udgjorde næsten en fjerdedel af de dyrkede *Campylobacter* species [23]. I et dansk studie fra Rigshospitalet af Aabenhus fra 2002 anvendte man filtermetoden på 11.500 fæcesprøver, og *C. concisus* var den hyppigste isolerede *Campylobacter* species med 110 ud af i alt 224 isolater [24].

I et endnu ikke publiceret studie fra Aalborg Sygehus er afføringsprøver, sendt til tarmbakteriologisk undersøgelse på Klinisk Mikrobiologisk Afd., blevet dyrket for *C. concisus* vha. filtermetoden. I løbet af 22 måneder, blev 10.388 afføringsprøver dyrket for *C. concisus*, ligesom de også blev dyrket på vanlig vis for andre tarmpatogene bakterier som *C. jejuni* og *Salmonella* species mm. Resultatet var, at der kunne dyrkes *C. concisus* fra 378 patienter med diarré. Dette blev kun overgået af *C. jejuni* med 456 patienter. Patienterne med *C. concisus* var hovedsageligt små børn og ældre. To tredjedele af patienterne var fra almen praksis, de resterende var fra et af regionens sygehuse. I ca. 10 % blev dyrkningen af *C. concisus*, ledsaget af en velkendt tarmpatogen såsom *Salmonella* species i afføringsprøven. Der er foreløbigt ikke klarhed over, om *C. concisus* er ligeså patogen, og om den kan give samme sygdomsforløb som *C. jejuni*, men der er en spørgeskema-undersøgelse i gang for at belyse dette.

Smittekilden og reservoiret for *C. concisus* er stadig uafklaret. I et australsk studie blev *C. concisus* dyrket i spytprøver i et meget højt antal fra både patienter og raske bærere [25]. Det er dog stadig uafklaret om menneskets mundhule eller mave-tarmkanal er reservoir for *C. concisus*, ligesom det er uvist om raske mundhule-bærere er i større risiko for at blive syge med diarré.

Der foreligger stadig ikke viden om, hvorvidt *C. concisus* er behandlingskrævende og om evt. antibiotisk behandling kan afkorte sygdomsforløbet hos patienter med diarré, hvor eneste dyrkningsfund i afføringsprøven er *C. concisus*. Et placebokontrolleret studie af behandlingseffekten ved *C. concisus* dyrkningspositiv diarré vil kunne give indsigt i patogeniciteten af *C. concisus* i vores del af verden. Det vil desuden belyse nødvendigheden af at diagnosticere en *C. concisus* infektion.

Formål

Formålet med dette studie er, at undersøge effekten af den antibiotiske behandling med azithromycin hos patienter med diarré hvor *C. concisus* er isoleret fra patientens afføringsprøve som den eneste mikroorganisme. Der må således ikke være en anden kendt tarmpatogen mikroorganisme som

konkurrerende årsag til patientens symptomer. Effekten bestemmes ud fra spørgeskemaer, der belyser patientens almene helbredstilstand samt udvikling i diarré-sygdom.
Parallelt gennemføres et studie med dyrkning af *C. concisus* fra spyt og afføring, før og efter behandling, for at belyse om eradikation af *C. concisus* er mulig ved behandling med azithromycin.

Hypoteser

- Azithromycin er placebo overlegent som antibiotisk behandling ved diarré forårsaget af *C. concisus* og kan afkorte sygdomsforløbet.

- Azithromycin er placebo overlegent til at eradikere *C. concisus* i spyt og afføring.

Metode

Studiedesign

- Randomiseret, dobbelt blindet, placebo kontrolleret, parallel gruppe klinisk studie af behandlingseffekt ved *C. concisus* dyrkningspositiv diarré.
- Azithromycin/placebo vil blive administreret med en tablet af 500 mg dagligt i 3 dage.
- Inden behandling afgives en spytprøve.
- Der føres dagbog fra behandlingens start til 7 dage efter behandlingsophør. dvs. samlet observationsperiode bliver i alt på 10 dage.
- Der indsendes én afføringsprøve samt spytprøve 7 dage efter behandlingens ophør, og igen efter 30 dage.
- Ved fortsatte diarré symptomer 10 dage efter studiestart, tilbydes patienten en ny klinisk vurdering, samt "cross-over" medicin. Her påbegyndes igen en ny observationsperiode på endnu 10 dage, hvorefter patienten på ny indsender af afføringsprøve og spytprøve.

Endepunkter

Endepunktet er "ændring i daglige antal afføringer", og sygdomsvarighed.
Effekten måles ved spørgeskemaer inklusiv "diarré-dagbog".

Det primære endepunkt:

- Antal dage til opnåelse af symptomfrihed = "normal afføringsfrekvens", tolket som < 3 afføringer/dag.

Sekundære endepunkter er:

- Antallet af daglige afføringer, indtil opnåelse af symptomfrihed.

- Forekomst af sekundære symptomer i form af mavesmerter, kvalme og opkastning

- Forekomst af bivirkninger sammenlignet med placebogruppen.

- Eradikation af *C. concisus* fra spyt og afføring.

Valg af azithromycin som antibiotisk behandling
Azithromycin tilhører antibiotika fra makrolid-gruppen, som er vidtgående ugiftige. Som tidligere nævnt er *C. jejuni* diarré oftest selvlimiterende, men behandling med et makrolid kan være indiceret i alvorlige tilfælde. Der findes flere forskellige slags makrolid antibiotika, og selv om der ikke er stor videnskabelig dokumentation herfor er azithromycin oftest førstevalg i USA og Europa [26-33]. (http://www.cdc.gov/nczved/divisions/dfbmd/diseases/campylobacter/).
Azithromycin har også den fordel at det kan administreres med en tablet dagligt, som kan øge patientens compliance. Almindelig *C. jejuni* har heller ikke samme resistens til makrolider, som til antibiotika af quinolon-gruppen, der ellers bruges i vid udstrækning til empirisk behandling af alvorlig diarré inkl. rejsediarré [34]. Resultaterne fra dyrkning af *C. concisus* i afføringsprøver på Klinisk Mikrobiologisk Afd. Aalborg Sygehus viser også, at *C. concisus* indtil nu har vist sig følsom til azithromycin. Det har også vist sig, at ca. en sjettedel af alle *C. concisus* isolater er resistente til antibiotika i quinolon-gruppen, hvorfor azithromycin må anses som det bedste valg af studie-antibiotikum.

Farmakokinetik af azithromycin
Nedenstående er i hht. www.medicin.dk
Azithromycin er syrestabilt. Efter oral indgift er biotilgængeligheden ca. 35 %. Efter indgift af 500 mg nås maksimal plasmakoncentration på 0,4 mikrogram/ml i løbet af 2-3 timer. Azithromycin fordeler sig hurtigt i organismen. Det trænger ind i cellerne og ophobes intracellulært, især i leukocytter og makrofager, hvor koncentrationen kan blive op til 100 gange plasmakoncentrationen. Fra disse celler udskilles det langsomt til interstitielrummet. Fordelingsvolumen er derfor stort (20-30 l/kg). Ved gentagen indgift af 500 mg dgl. i 3-5 dage stiger den maksimale plasmakoncentration kun til 0,6 mikrogram/ml. Passerer ikke blod-hjernebarrieren. Terminal plasmahalveringstid ca. 40 timer. Ca. 50 % udskilles uomdannet med galden og ca. 12 % gennem nyrerne.

Randomisering
Hver patient får tildelt et studienummer og randomiseres til forsøgsmedicin/placebo i forholdet 1:1. **Glostrup apotek v. apoteker Steen Mejlgaard, varetager mærkning og pakning af forsøgsmedicinen (www.glostrup-apotek.dk). Randomiserings koden opbevares på forsøgscentret (i Trial Master File)** og vil først blive brudt ved afslutning af studiet, med mindre særlige omstændigheder, som for eksempel mistanke om alvorlige bivirkninger, gør tidligere kodebrud påkrævet. **Ved behov for oplysning om det enkelte randomiseringsnummers sammensætning under projektforløbet, kan det oplyses af Glostrup apotek via tlf. 43 96 00 20, der har åbent hele døgnet.** Både patient og den projektansvarlige læge er uvidende om hvilken behandlingsgruppe den enkelte patient er randomiseret til. Til hvert studienummer er der pakket "Cross-over"-medicin, til brug ved fortsatte symptomer efter 10 dage.

Tilrettelæggelse
Studieperioden er fra **1. april 2012** til **31. november 2013**.

Kun patienter over 18 år kan deltage. Patienterne skal være bosiddende i Region Nordjylland. Patienterne inkluderes blandt patienter, der findes *C. concisus* positive på Klinisk Mikrobiologisk Afd., Aalborg Sygehus. I patientens afføringsprøve må ingen anden tarmpatogen mikroorganisme være blevet identificeret.
I studieperioden undersøges alle afføringsprøver fra voksne patienter for *C. concisus*.
Patienterne kan være fra både almen praksis samt fra et af sygehusene i Region Nordjylland.

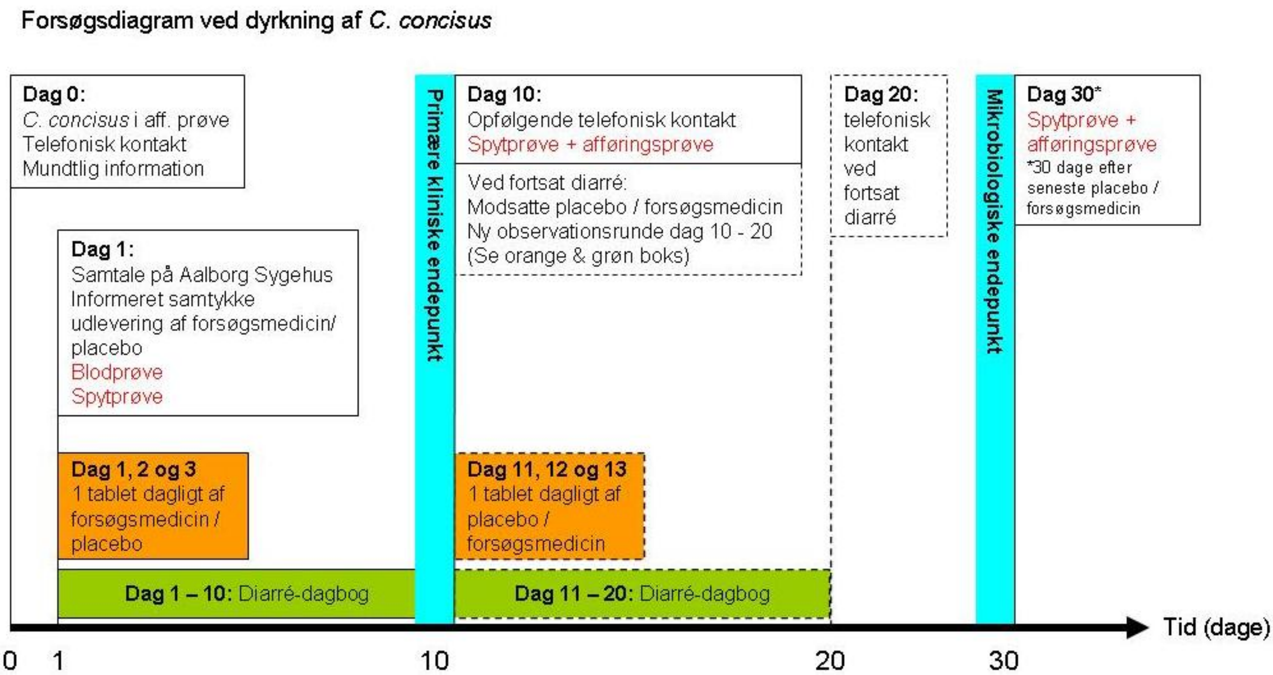

- **Information til rekvirerende/behandlende læge**

Inden inkludering af forsøgspersonen i forsøget har denne haft lægelig kontakt, der førte til rekvirering af en tarmbakteriologisk undersøgelse af patientens afføring. Hvad enten det er patientens praktiserende læge eller en hospitalslæge, der bestilte undersøgelsen, **bliver rekvirerende/behandlende læge informeret mundtligt om forsøget telefonisk af enten sponsorinvestigator eller af en subinvestigator.** Den mundtlige besked bliver fulgt op af en skriftlig besked til den rekvirerende læge. Hvad angår de praktiserende læger, så informeres de via en skrivelse via praksiskonsulent, praktiserende læge Jan Nybo.

- **1. kontakt til patienten.**

Efter at mundtlig og skriftlig information er givet til den rekvirerende/behandlende læge, og at denne giver mundtlig accept hertil, vil patienten herefter blive kontaktet telefonisk af sponsorinvestigator eller af en subinvestigator. Der gives en grundig mundtligt information om dyrkningsfundet af *C. concisus* i patientens afføringsprøve. Hvis patienten fortsat har diarré og der ikke er identificeret en anden patogen mikroorganisme, vil patienten, blive indbudt til samtale med den projektansvarlige læge. Der vil desuden blive oplyst om antal dage, som projektet strækker sig over og patienten informeres også om, at det er muligt at medbringe en bisidder i forbindelse med samtalen.

- **1. kliniske besøg mellem patient og den projektansvarlige læge.**

Samtalen vil foregå i uforstyrrede omgivelser i et samtalerum i infektionsmedicinsk afdeling på Aalborg Sygehus. Patienten vil få grundig mundtlig og skriftlig information om studiet, og der vil blive gjort rede for bivirkninger fra medicinen mm. Patienterne vil få udleveret:

- Den Centrale Videnskabsetiske Komité's skrivelse: "Forsøgspersonens rettigheder i et biomedicinsk forskningsprojekt".
- Skriftlig deltagerinformation.
- **Prøvetagningsvejledning.**

- **Kopi til eget brug af informeret samtykke.**
- **Kopi til eget brug af fuldmagtserklæring**.

Der indhentes informerende samtykke **og fuldmagtserklæring** ved samtalen efter der har været tid til at læse det udleverede skriftlige materiale igennem. Patienten har ret til betænkningstid og til at medbringe en bisidder ved informationssamtalen. Patienten underskriver og daterer både samtykkeerklæringen **og fuldmagtserklæringen** i **hver** to eksemplarer og beholder selv et eksemplar **af hver**. Den projektansvarlige læge attesterer samtidigt, at mundtlig information har fundet sted og at skriftlig information er udleveret. Hvis patienten giver skriftligt samtykke vil patienten blive behandlet 3 dage med tablet, azithomycin, 500mg eller placebo, med en tablet dagligt i 3 dage. Placebo vil være i en tablet af samme udseende, størrelse og lugt som tabletten indeholdende azithromycin.

Etikette til både azithromycin og placebo forfattes på dansk og vil indeholde:

1- Til klinisk forsøg
2- At indholdet er tabletter samt antallet af tabletter i beholderne.
3- Kodebetegnelse - i form af studienummer.
4- Batchnummerangivelse som tillader opretholdelse af blinding
5- Fabrikant
6- Udløbsdato
7- Dosering: 1 tablet x 1 dagligt, i 3 dage
8- Den forsøgsansvarliges navn: Hans Linde Nielsen, Infektionsmedicinsk afdeling, Aalborg Sygehus, Tlf.: 99 32 65 32, samt e-mail: halin@rn.dk

Patienten informeres om, at der ikke må indtages ”stoppende”-medicin i form af Loperamid, under studieperioden.

Til samtalen vil patienten blive informeret om udfyldelse af i alt 2 spørgeskemaer:

1- Første spørgeskema udfyldes før opstart af behandling. Det vedrører patientens almene helbredsoplysninger, samt dato for og symptomer på diarré-sygdom.
2- Andet spørgeskema skal udfyldes løbende de næste 10 dage. Patienten skal dagligt føre dagbog om diarré-symptomer, og dermed udvikling herpå. Dvs. patienten skal notere forløbet under og efter de 3 dages behandling.

Til spørgeskemaet udleveres frankerede svarkuverter.

Ved samtalen vil patienten blive adspurgt om at afgive en spytprøve. Desuden vil patienten blive bedt om at indsende en spytprøve og afføringsprøve 10 dage efter 1. besøg.
Der vil også blive udleveret utensilier herpå, samt en prøvetagningsvejledning.

Til 1. besøg vil patienten også blive adspurgt om afgivelse af en blodprøve til måling af væsketal og infektionstal.

Gennem udfyldelse af spørgeskema indhentes der oplysninger om patienternes kliniske forløb og evt. bivirkninger til behandlingen.

10 dage efter behandlingen, kontaktes patienten telefonisk igen af den projektansvarlige læge med henblik på et telefoninterview vedrørende monitorering af bivirkninger, samt patientens sygdomsforløb. Hvis patienten 10 dage efter behandling forsat er svært generet af diarré, tilbydes et 2. besøg, på infektionsmedicinsk afd. Her tilbydes ”Cross-over” medicin, med efterfølgende observationsperiode på 10 dage. Dvs. Hvis patienten initialt har fået placebo, kan behandling med

azithromycin påbegyndes efter 10 dage. Ligeså, hvis patienten initialt har fået azithromycin kan patienten behandles med placebo 10 dage efter. **"Cross-over" medicinen er dog også blindet for både investigator og forsøgsperson.**
Efter "cross-over" medicin, og efterfølgende observationsperiode i endnu 10 dage, fortages andet telefoninterview på 10 dagen. Ved fortsatte symptomer på diarré-sygdom, 20 dage efter studiestart, vurderer den projektansvarlige læge, muligheden for yderligere behandlingstiltag evt. via patientens praktiserende læge, eller via henvisning til speciale afdeling.

Patienten indsender på ny en afføringsprøve og spytprøve efter 20 dages observationsperiode. Afslutningsvis indsender patienten endnu et sæt afføringsprøve og spytprøve 30 dage efter 1. besøg. Hvis patienten har modtaget "Cross-over" medicin skal prøverne indsendes 30 dage herefter.

Compliance
Forsøgspersonen skal indtage 100 % af forsøgsmedicinen i løbet af studie perioden. Compliance vurderes dels ved interview, dels ved optælling af udleveret og tilbageleveret forsøgsmedicin.

Bivirkninger, risici og ulemper
Antibiotika af makrolid-gruppen og dermed azithromycin, er vidtgående ugiftige. De hyppigste bivirkninger er madlede, kvalme, abdominalsmerter og forbigående diarré (Se nedenstående tabel). Azithromycin er det antibiotikum, i makrolid-gruppen, der har den bedste bivirkningsprofil.

Bivirkninger og hændelser som finder sted under forsøget registreres af patienten i udleveret dagbog og gennemgås sammen med den projektansvarlige læge, der også er sponsorinvestigator på projektet. Såfremt eventuelle bivirkninger bliver utålelige eller udgør en helbredsmæssig risiko vil patienten omgående udgå af studiet og relevante tiltag vil blive iværksat med henblik på videre opfølgning og behandling.

Efter forsøgsledelsens opfattelse er der således ikke forbundet nogen kendt risiko ved at deltage i studiet, når den beskrevne metode følges og de beskrevne forholdsregler iagttages.

I nedenstående tabel er alle bivirkninger, der er identificeret via klinisk erfaring og overvågning efter markedsføringen, angivet efter organklasse og hyppighed.
Frekvensgruppen er defineret i henhold til følgende konvention:

- Meget almindelig (≥ 1/10),
- Almindelig (≥ 1/100 til < 1/10)
- Ikke almindelig (≥ 1/1.000 til < 1/100)
- Sjælden (≥ 1/10.000 til < 1/1.000)
- Meget sjælden (< 1/10.000)
- Ikke kendt (kan ikke estimeres ud fra forhåndenværende data).

Inden for hver enkelt frekvensgruppe er bivirkningerne opstillet efter, hvor alvorlige de er. De alvorligste bivirkninger er anført først.

System-organklasse	Hyppighed	Bivirkning
Infektioner og parasitære sygdomme	Ikke almindelig	Candidiasis,<br>oral candidiasis, vaginal infektion
	Ikke kendt	Pseudomembranøs colitis

Blod og lymfesystem	Almindelig	Nedsat lymfocyttal, øget eosinofiltal
	Ikke almindelig	Leukopeni, neutropeni
	Sjælden	Trombocytopeni, hæmolytisk anæmi
Immunsystemet	Ikke almindelig	Angioødem, overfølsomhed
	Ikke kendt	Anafylaktisk reaktion
Metabolisme og ernæring	Almindelig	Anoreksi
Psykiske forstyrrelser	Ikke almindelig	Nervøsitet
	Sjælden	Agitation, depersonalisation
	Ikke kendt	Aggression, angst
Nervesystemet	Almindelig	Svimmelhed, hovedpine, paræstesi, dysgeusi
	Ikke almindelig	Hypæstesi, somnolens, insomni
	Ikke kendt	Synkope, kramper, psykomotorisk hyperaktivitet, anosmi, ageusi, parosmi, myasthenia gravis
Øjne	Almindelig	Nedsat syn
Øre og labyrint	Almindelig	Døvhed
	Ikke almindelig	Nedsat hørelse, tinnitus
	Sjælden	Vertigo
Hjerte	Ikke almindelig	Palpitationer
	Ikke kendt	Torsades de pointes, arytmi herunder ventrikulær takykardi, forlænget QT-interval på elektrokardiogram
Vaskulære sygdomme	Ikke kendt	Hypotension
Mave-tarm-kanalen	Meget almindelig	Diarré, mavesmerter, kvalme, flatulens
	Almindelig	Opkastning, dyspepsi
	Ikke almindelig	Gastritis, forstoppelse
	Ikke kendt	Pancreatitis, misfarvning af tungen
Lever og galdeveje	Ikke almindelig	Hepatitis, forhøjet niveau af aspartat-amino-transferase, forhøjet niveau af alanin-amino-transferase, forhøjet niveau af bilirubin i blodet
	Sjælden	Unormal leverfunktion
	Ikke kendt	Leversvigt (fulminant hepatitis, levernekrose, kolestatisk icterus)
Hud og subkutane væv	Almindelig	Udslæt, pruritus
	Ikke almindelig	Stevens-Johnsons syndrom, lysfølsomhedsreaktion, urticaria
	Ikke kendt	Toksisk epidermal nekrolyse,

		erythema multiforme
Knogler, led, muskler og bindevæv	Almindelig	Artralgi
Nyrer og urinveje	Ikke almindelig	Øget niveau af urinstof i blodet
	Sjælden	Akut nyresvigt, interstitiel nefritis
Almene symptomer og reaktioner på administrations-stedet	Almindelig	Træthed
	Ikke almindelig	Smerter i brystet, ødem, utilpashed, asteni
Undersøgelser	Almindelig	Fald i niveauet af bicarbonat i blodet
	Ikke almindelig	Abnormt niveau af kalium i blodet

Indberetninger til Lægemiddelstyrelsen
Sponsorinvestigator vil omgående underrette Lægemiddelstyrelsen, hvis der under forsøget optræder uventede og alvorlige formodede bivirkninger (Suspected Unexpected Serious Adverse Reactions, SUSARs) jf. lægemiddelloven § 89 stk. 2. nr. 1.
Definitionerne er som følger jf. bekendtgørelsen for kliniske forsøg:

- hændelse: enhver uønsket hændelse hos en patient eller en forsøgsperson i et klinisk forsøg efter behandling med et lægemiddel, uden at der nødvendigvis er sammenhæng mellem denne behandling og den uønskede hændelse
- bivirkning: enhver skadelig og uønsket reaktion på et forsøgslægemiddel uanset dosis
- uventet bivirkning: en bivirkning, hvis karakter eller alvor ikke stemmer overens med produktoplysningerne (fx Investigators-Brochuren for et ikke-godkendt forsøgslægemiddel eller produktresuméet, hvis der er tale om et godkendt produkt)
- alvorlig hændelse eller alvorlig bivirkning: en hændelse eller bivirkning, som uanset dosis resulterer i død, er livstruende, medfører hospitalsindlæggelse eller forlængelse af hospitalsophold, resulterer i betydelig eller vedvarende invaliditet eller uarbejdsdygtighed eller fører til en medfødt anomali eller misdannelse.

Sponsorinvestigator registrerer og indberetter alle oplysninger om SUSARs, som er dødelige eller livstruende, til Lægemiddelstyrelsen hurtigst muligt og senest 7 dage efter, at sponsorinvestigator har fået kendskab til en sådan formodet bivirkning.
Sponsorinvestigator vil senest 8 dage efter indberetningen meddele Lægemiddelstyrelsen om alle relevante oplysninger om sponsorsinvestigators opfølgning på indberetningen.

Alle andre SUSAR, vil blive indberettet af sponsorinvestigator til Lægemiddelstyrelsen senest 15 dage efter, at sponsorinvestigator har fået kendskab til disse.

Indberetning vil foregå på lægemiddelsstyrelsens hjemmeside på følgende link:

http://laegemiddelstyrelsen.dk/da/topics/bivirkninger-og-forsoeg/bivirkninger/meld-en-bivirkning-eller-utilsigtet-haendelse/kliniske-forsoeg/indberetning-af-mistaenkte-uventede-og-a---e-blanket

Følgende produktresume vil blive anvendt, når det skal vurderes om en alvorlig relateret bivirkning (SAR) er uventet/ventet, og dermed muligvis bliver en SUSAR.

http://www.produktresume.dk/docushare/dsweb/Get/Document-27937/Azithromycin+2care4+%282care4%29%2C+filmovertrukne+tabletter+500+mg.doc

Guideline fra EU vil også blive fulgt
(Detailed guidance on the collection, verification and presentation of adverse event/reaction reports arising from clinical trials on medicinal products for human use ('CT-3'))

Direkte link:
http://ec.europa.eu/health/files/eudralex/vol-10/2011_c172_01/2011_c172_01_en.pdf

Forsøgspersonerne afsluttes dag 10 (evt. dag 20 ved "Cross-over) efter telefonsamtale med sponsorinvestigator (se forsøgsdiagram). Sponsorinvestigator står dog til rådighed for forsøgspersonerne i hele studieperioden fra dag 0 til dag 30 (sidste afføringsprøve), og kan kontaktes løbende på telefon og e-mail.

Forsøgspersoner
I alt ønskes et minimum af 100 patienter inkluderet med 50 patienter i placebogruppen og 50 patienter i behandlingsgruppen. Patienter, der skønnes at droppe ud af studiet under forsøget er sat til 10 % i hver gruppe. Tidsrammen kan eventuelt udvides for at opnå tilsigtede antal deltagere.

Antal patienter til indgang i studiet er beregnet på baggrund af følgende antagelser.
Type 1 fejl, 2a=0,05
Type 2 fejl, b=0,20
Forventet effekt af placebo: 0,20
Forventet effekt af azithromycin: 0,60
Statistisk power: 80

Antallet er beregnet vha. programmet: STATA vha. kommandoen "db sampsi".
Antagelser:

- Gennemsnits varighed af diarre i placebo gruppen = 7
- Standard Deviation i placebo gruppen = 5
- Gennemsnits varighed af diarre i azithromycin gruppen = 4
- Standard Deviation i azithromycin gruppen = 5
- n(placebo)/n(azithromycin) = 1

n(placebo) =44
n(azithromycin) =44

Inklusionkriterier

- Diarré patienter over 18 år med positiv *C. concisus* dyrkning fra fæces.
- Fortsat diarré defineret som 3 eller flere vandtynde til grødede afføringer dagligt
eller
- 2 vandtynde til grødede afføringer, samt minimum et eller flere ledsagesymptomer, såsom mavesmerter, kvalme, opkastning eller feber.
- Symptomer igennem minimum 24 timer.

- **Symptomdebut på maximalt 21 dage.**
- Mundligt og skriftligt samtykke, med dokumentation for at alle relevante oplysninger om studiet er givet til patienten.
- Patienten skal være villig til og have mulighed for at møde til det planlagte besøg og overholde den planlagte studieplan.

Eksklusionskriterier

- Overfølsomhed over for azithromycin, erythromycin, makrolid- eller ketolidantibiotika eller over for et eller flere af hjælpestofferne.
- Alder under 18.
- Graviditet eller amning.
- Positiv afføringsprøve for anden tarmpatogen mikroorganisme.
- Behandling med andet antibiotikum (på et hvilket som helst tidspunkt fra 14 dage før første afføringsprøve).
- **Patienter med kolostomi og ileostomi.**
- Patienter med alvorlig leversygdom.
- Patienter med alvorlig nedsat nyrefunktion (GFR < 10 ml/min).
- patienter med kongenit eller dokumenteret erhvervet forlænget QT-interval.
- Patienter i behandling med andre aktive stoffer, der forlænger QT-intervallet som f.eks. antiarytmika i klasse IA og III, cisaprid og terfenadin.
- Patienter med elektrolytforstyrrelser, specielt hypokaliæmi og hypomagnesæmi.
- Patienter med klinisk relevant bradykardi, hjertearytmi eller alvorlig hjerteinsufficiens.
- Kronisk inflammatorisk tarmsygdom.
- Kronisk diare af anden kendt årsag.
- Demens.
- **Alvorlig sygdom mindre end 21 dage fra planlagt indgang i studiet.**
- Patienten er i behandling med medicin, der har interaktion med azithromycin f.eks. sekalealkaloider, ciclosporin eller amiodaron.
- Patienter involveret i planlægning eller udførelse af studiet.

Relevante tarmpatogene bakterier, virus og parasitter

Bakterier: *Salmonella, Shigella, Yersinia, Campylobacter jejuni, , Aeromonas, Plesiomonas, Vibrio, Clostridium difficile, E. coli* (EHEC, VTEC) EPEC, A/EEC, EAggEC, EIEC), Virus: Norovirus, Rotavirus, enteriske adenovirus, Parasitter: *Giardia lamblia* og *Cryptosporidium parvum*.
Fæces gemmes til både virus og parasitundersøgelse på Klinisk Mikrobiologisk Afd. Aalborg Sygehus eller Statens Serum Institut:

- ca. 200 mg fæces fryses i oksebouillon med glycerol 10 % ved -80°C til senere brug.
  Røret mærkes med nr samt frysenr på låget og sættes i fryseæske (SSI varenr. 75699) mærkes med "Fæces: C. concisus & Azithromycin". Prøverne sættes i den projektansvarlige læges fryseplads på Klinisk Mikrobiologisk Afd. Aalborg Sygehus.
- ca. 200 mg gemmes i tomt 2ml eppendorf rør (SSI varenr 5998) (-20°C til senere virus og parasit undersøgelser). Prøverne sættes i den projektansvarlige læges fryseplads på Klinisk Mikrobiologisk Afd. Aalborg Sygehus.

Indsamling og håndtering af data
Oplysninger om forsøgspersonen beskyttes efter lov om behandling af personoplysninger og sundhedsloven og der indhentes tilladelse til brug af patientrelaterede data (journaloplysninger fra indlagte patienter og laboratoriesvar) samt opbevaring af data fra datatilsynet. Dataindsamling og håndtering vil foregå efter GCP's retningslinier, ligesom studiet udføres efter CONSORT rekommandationerne.
http://www.consort-statement.org/
Forsøget vil blive forhåndsgodkendt og monitoreret af GCP-enheden ved Aarhus Universitet.
http://gcp.au.dk/
Forsøget vil også blive registreret i den amerikanske database:
http://www.clinicaltrials.gov/

Økonomiske aspekter
Projektet er en videreførelse af et afsluttet ph.d. projekt, og den projektansvarlige læge fortsætter sin nuværende forskningsstilling og vil blive aflønnet i forhold til dette. Supplerende finansiering vil blive søgt i nationale og internationale fonde. Patienter indgår uden økonomisk kompensation, men kan få transportudgifter dækket.

Etiske aspekter
Diarre af enhver årsag er forbundet med betydelige gener for den enkelte patient, og har betydelige økonomiske omkostninger for den enkelte patient, men også for samfundet i forbindelse med sygemeldinger og evt. undersøgelser og behandling. En nærmere belysning af årsager til infektiøs diarre kan føre til bedre forebyggelse og behandling. Patienter behandles med velkendt antibiotika (azithromycin), der har været brugt i mange år i Danmark bl.a. til behandling af Klamydia.
Formålet med denne undersøgelse er således, at belyse den ætiologiske betydning af *C. concisus* som årsag til diarré, og vurdere effekten af behandling af infektionen med denne bakterie. På den baggrund er det nødvendigt at foretage en placebokontrolleret undersøgelse.
Azithromycin er et præparat der rutinemæssigt anvendes til bakterielle infektioner, såsom Klamydia. Bivirkninger ved behandlingen er få, men kan forekomme, se tabel 1. Behandlingen kan muligvis gavne de patienter der efter lodtrækning modtager azithromycin behandling, og det kan komme fremtidige patienter til gavn.
Undersøgelsen overholder kravene i Helsinki deklarationen (5. udgave), og forelægges den Videnskabsetiske Komité Region Nordjylland samt Lægemiddelstyrelsen. Desuden anmeldes forsøget til datatilsynet og monitoreres af GCP-enheden ved Aarhus Universitet.
Deltagelse i undersøgelsen er frivillig og indhentede oplysninger vil blive behandlet fortroligt, og deltagende personer kan til enhver tid vælge at trække sig ud af undersøgelsen. Informeret samtykke vil blive indhentet fra patienten. Afføringsprøver (få ml) der ikke umiddelbart anvendes i projektet, opbevares nedfrosset på Klinisk Mikrobiologisk Afd., Aalborg Sygehus med henblik på evt. yderligere forskning i tilfælde af at nye metoder udvikles mm. Fæcesprøverne vil blive opbevaret og analyseret i Danmark, og vil ikke blive videreført til udlandet. De vil blive opbevaret i maximalt 5 år, hvorefter de vil blive destrueret. Nye projekter vil blive anmeldt til den regionale videnskabsetiske komite, og deltagerne anmodes på forhånd om tage stilling til, om de ønsker at få oplysning om resultater herfra, der kan have betydning for udvikling af sygdomme eller behandling. **Spytprøver vil ikke blive nedfrosset.**
Det skønnes, at opnåelsen af denne viden står i rimeligt forhold til de skitserede risici og ulemper patienterne udsættes for. Som led i projektet skal patienterne desuden aflevere en ekstra afføringsprøve. Dette indebærer fraset ubehaget ingen risici for patienten. I forbindelse med blodprøvetagning kan der forekomme let ubehag og der kan forekomme en lille blodansamling ved stik gennem blodåren. Desuden er der en minimal risiko for infektion i huden og lokal irritation ved prøvetagningsstedet.

Spørgeskemaet er valideret fra andre undersøgelser af kroniske tarmsymptomer og generelle psykiske symptomer. Det vurderes ikke at spørgeskemaet kan være psykisk belastende at besvare, eller kan give varige psykiske mén. Det skønnes ikke at projektet rummer en interessekonflikt mellem erhvervelse af ny viden og samfunds- eller økonomiske interesser.

Publikation

Resultaterne fra undersøgelsen vil blive offentliggjort på nationale og internationale møder samt publiceret i internationale peer-reviewede tidsskrifter. Både positive som negative resultater vil blive publiceret. Alle publikationsrettigheder tilfalder investigator. Forfatterrækkefølgen betinges herudover af den enkeltes indsats i forhold til publikationen.

Reference List

(1) Wingstrand A, Neimann J, Engberg J, Nielsen EM, Gerner-Smidt P, Wegener HC, et al. Fresh chicken as main risk factor for campylobacteriosis, Denmark. *Emerg Infect Dis* 2006; 12: 280-285.

(2) Zilbauer M, Dorrell N, Wren BW, Bajaj-Elliott M. Campylobacter jejuni-mediated disease pathogenesis: an update. *Trans R Soc Trop Med Hyg* 2008; 102: 123-129.

(3) Engberg J. Contributions to the epidemiology of Campylobacter infections. A review of clinical and microbiological studies. *Dan Med Bull* 2006; 53: 361-389.

(4) Andersen LP, Gerner-Smidt P, Jensen TE, Jensen KT, Lester A, Tvede M. Anbefalinger fra DSKM's arbejdsgruppe for tarmbakteriologi. 2003.
Ref Type: Generic

(5) Vandenberg O, Dediste A, Houf K, Ibekwem S, Souayah H, Cadranel S, et al. Arcobacter species in humans. *Emerg Infect Dis* 2004; 10: 1863-1867.

(6) S.Ethelberg, K.Mølbak, K.E.P.Olsen, F.Scheutz. Zoonotiske Tarminfektioner 2009. 2010.

(7) Ethelberg S, Olsen KE, Gerner-Smidt P, Molbak K. The significance of the number of submitted samples and patient-related factors for faecal bacterial diagnostics. *Clin Microbiol Infect* 2007; 13: 1095-1099.

(8) Engberg J. Contributions to the epidemiology of Campylobacter infections. A review of clinical and microbiological studies. *Dan Med Bull* 2006; 53: 361-389.

(9) Poly F, Guerry P. Pathogenesis of Campylobacter. *Curr Opin Gastroenterol* 2008; 24: 27-31.

(10) Zilbauer M, Dorrell N, Wren BW, Bajaj-Elliott M. Campylobacter jejuni-mediated disease pathogenesis: an update. *Trans R Soc Trop Med Hyg* 2008; 102: 123-129.

(11) Hansen NE, Haunsø S, Schaffalitzky De Muckadell OB. Medicinsk Kompendium, 16. udgave, 2004. 16. udgave ed. NYT NORDISK FORLAG ARNOLD BUSCK A/S 2004; 2004.

(12) Spiller RC. Role of infection in irritable bowel syndrome. *J Gastroenterol* 2007; 42 Suppl 17: 41-47.

(13) Talley NJ, Spiller R. Irritable bowel syndrome: a little understood organic bowel disease? *Lancet* 2002; 360: 555-564.

(14) Talley NJ. Irritable bowel syndrome. *Intern Med J* 2006; 36: 724-728.

(15) Spiller RC. Is IBS caused by infectious diarrhea? *Nat Clin Pract Gastroenterol Hepatol* 2007; 4: 642-643.

(16) Drossman DA. The functional gastrointestinal disorders and the Rome III process. *Gastroenterology* 2006; 130: 1377-1390.

(17) Townes JM, Deodhar AA, Laine ES, Smith K, Krug HE, Barkhuizen A, et al. Reactive arthritis following culture-confirmed infections with bacterial enteric pathogens in Minnesota and Oregon: a population-based study. *Ann Rheum Dis* 2008.

(18) Engberg JH. [Guillain-Barre syndrome and Campylobacter]. *Ugeskr Laeger* 2002; 164: 5905-5908.

(19) Hughes RA, Cornblath DR. Guillain-Barre syndrome. *Lancet* 2005; 366: 1653-1666.

(20) Engberg J, On SL, Harrington CS, Gerner-Smidt P. Prevalence of Campylobacter, Arcobacter, Helicobacter, and Sutterella spp. in human fecal samples as estimated by a reevaluation of isolation methods for Campylobacters. *J Clin Microbiol* 2000; 38: 286-291.

(21) Chaban B, Musil KM, Himsworth CG, Hill JE. Development of cpn60-based real-time quantitative PCR assays for the detection of 14 Campylobacter species and application to screening of canine fecal samples. *Appl Environ Microbiol* 2009; 75: 3055-3061.

(22) Lindblom GB, Sjogren E, Hansson-Westerberg J, Kaijser B. Campylobacter upsaliensis, C. sputorum sputorum and C. concisus as common causes of diarrhoea in Swedish children. *Scand J Infect Dis* 1995; 27: 187-188.

(23) Lastovica AJ. Emerging Campylobacter spp.: The tip of the iceberg. *Clinical Microbiology Newsletter - 1 April 2006 (Vol 28, Issue 7, Pages 49-56, DOI: 10 1016/j clinmicnews 2006 03 004)* 2010.

(24) Aabenhus R, Permin H, On SL, Andersen LP. Prevalence of Campylobacter concisus in diarrhoea of immunocompromised patients. *Scand J Infect Dis* 2002; 34: 248-252.

(25) Zhang L, Budiman V, Day AS, Mitchell H, Lemberg DA, Riordan SM, et al. Isolation and detection of Campylobacter concisus from saliva of healthy individuals and patients with inflammatory bowel disease. *J Clin Microbiol* 2010; 48: 2965-2967.

(26) Gendrel D, Cohen R. [Bacterial diarrheas and antibiotics: European recommendations]. *Arch Pediatr* 2008; 15 Suppl 2: S93-S96.

(27) Vukelic D, Trkulja V, Salkovic-Petrisic M. Single oral dose of azithromycin versus 5 days of oral erythromycin or no antibiotic in treatment of campylobacter enterocolitis in children: a prospective randomized assessor-blind study. *J Pediatr Gastroenterol Nutr* 2010; 50: 404-410.

(28) Jones K, Felmingham D, Ridgway G. In vitro activity of azithromycin (CP-62,993), a novel macrolide, against enteric pathogens. *Drugs Exp Clin Res* 1988; 14: 613-615.

(29) Hill DR, Ericsson CD, Pearson RD, Keystone JS, Freedman DO, Kozarsky PE, et al. The practice of travel medicine: guidelines by the Infectious Diseases Society of America. *Clin Infect Dis* 2006; 43: 1499-1539.

(30) Hill DR, Beeching NJ. Travelers' diarrhea. *Curr Opin Infect Dis* 2010; 23: 481-487.

(31) Sanders JW, Frenck RW, Putnam SD, Riddle MS, Johnston JR, Ulukan S, et al. Azithromycin and loperamide are comparable to levofloxacin and loperamide for the treatment of traveler's diarrhea in United States military personnel in Turkey. *Clin Infect Dis* 2007; 45: 294-301.

(32) Adachi JA, Ericsson CD, Jiang ZD, DuPont MW, Martinez-Sandoval F, Knirsch C, et al. Azithromycin found to be comparable to levofloxacin for the treatment of US travelers with acute diarrhea acquired in Mexico. *Clin Infect Dis* 2003; 37: 1165-1171.

(33) Tribble DR, Sanders JW, Pang LW, Mason C, Pitarangsi C, Baqar S, et al. Traveler's diarrhea in Thailand: randomized, double-blind trial comparing single-dose and 3-day azithromycin-based regimens with a 3-day levofloxacin regimen. *Clin Infect Dis* 2007; 44: 338-346.

(34) Engberg J, Aarestrup FM, Taylor DE, Gerner-Smidt P, Nachamkin I. Quinolone and macrolide resistance in Campylobacter jejuni and C. coli: resistance mechanisms and trends in human isolates. *Emerg Infect Dis* 2001; 7: 24-34.

Lægmandsresume

Campylobacter concisus placebokontrolleret behandlingsstudie

Baggrund

Det er almindeligt forekommende at blive syg på grund af smitte med bakterier fra fødevarer. Symptomerne på dette er oftest opkastning og diarré. Den hyppigste årsag til denne infektion i Danmark er bakterien *Campylobacter*. De fleste gange forsvinder sygdommen af sig selv igen, men længden af sygdommen og alvoren af den varierer fra person til person. Det er velkendt, at der er 2 *Campylobacter* arter, der kan give diarré-sygdom, men en ny undersøgelse på Aalborg Sygehus, har imidlertid vist at der findes en tredje art af *Campylobacter* bakterien (kaldet *Campylobacter concisus*), som forekommer næsten ligeså hyppigt som de velkendte *Campylobacter* arter. Symptombilledet for denne *Campylobacter* art er formentligt også opkastning, diarré og feber. Infektionen kan optræde i alle aldre, men vores viden om, hvor bakterien kommer fra, og om patienterne kan have effekt af antibiotisk behandling er meget begrænset.

Undersøgelsens formål

Formålet med undersøgelsen er derfor at undersøge om antibiotisk behandling af *C. concisus* bakterier kan bedre diarré tilstanden og afkorte varigheden.

Forsøgsdeltagere

Patienterne inkluderes blandt voksne patienter, der findes *C. concisus* positive i Klinisk Mikrobiologisk Afd., Aalborg Sygehus og hvor ingen anden tarmpatogen mikroorganisme er blevet identificeret.

Metode

Studieperioden er fra 1. februar 2012 til **31. december 2013**. I studieperioden undersøges alle afføringsprøver sendt til tarmbakteriologisk undersøgelse på Klinisk Mikrobiologisk Afd. på Aalborg Sygehus for *C. concisus*. Undersøgelsen er designet som et placebokontrolleret behandlingsstudie blandt diarré patienter med dyrkningspositiv *C. concisus* infektion. Ved positiv dyrkning kontaktes patienten telefonisk af den projektansvarlige læge og hvis patienten fortsat har diarré, vil patienten blive indkaldt til samtale med den projektansvarlige læge. Det vil blive oplyst, at det er muligt at medbringe en bisidder i forbindelse med samtalen. Hvis der efter grundig mundtlig og skriftlig information gives skriftligt samtykke til deltagelse vil patienten blive behandlet 3 dage med antibiotika (azithromycin) eller placebo. Placebo vil være i en tablet af samme udseende, størrelse og lugt som tabletten indeholdende azithromycin. Ved samtalen vil patienten desuden blive bedt om at afgive en spytprøve samt en blodprøve. I forbindelse med blodprøvetagning kan der forekomme let ubehag og der kan forekomme en lille blodansamling ved stik gennem blodåren. Der udfyldes et spørgeskema før opstart af behandling vedr. almene helbredsoplysninger samt om den aktuelle sygdomsepisode. Der føres diarré-dagbog under behandling og i 7 dage efter endt behandling. dvs. i alt i 10 dage. Efter endt behandling foretages kontroldyrkning fra afføring og spyt, og den projektansvarlige læge har et telefoninterview med patienten. Hvis der fortsat er symptomer på diarré, tilbydes en ny samtale og ”cross-over”-behandling (hvis der initialt er givet placebo, så behandles med antibiotika i anden omgang, og omvendt) og en ny 10 dages observationsperiode følger herefter. **”Cross-over” medicinen er dog også blindet for både investigator og forsøgsperson.** Afslutningsvis indsendes endnu en afføringsprøve og en spytprøve 30 dage efter start af forsøgsmedicin/placebo. I alt ønskes 100 patienter inkluderet med 50 patienter i placebogruppen og 50 patienter i behandlingsgruppen. Tidsrammen kan eventuelt udvides for at opnå tilsigtede antal deltagere. Gennem udfyldelse af spørgeskema indhentes der oplysning om klinik og evt. bivirkninger til behandlingen.

Læge, Hans Linde Nielsen, Telefon: 99 32 65 32, E-mail: halin@rn.dk

Resultaterne offentliggøres i et lægevidenskabeligt tidsskrift eller andet tidsskrift med fuld anonymitet for patienten. Herefter vil materialet med patienternes personlige data blive destrueret. En afføringsprøve opbevares dog nedfrosset på afdelingens laboratorium. Hvis prøverne ønskes anvendt til et nyt forskningsprojekt, anmeldes dette projekt til etisk komité.

Bivirkninger, risici og ulemper
Som alle andre lægemidler kan azithromycin i anbefalede doser have bivirkninger.
Azithromycin er dog et af de hyppigste anvendte antibiotika, som den praktiserende læge bruger til behandling af infektioner hos både børn og voksne.

Tabel 1. Behandling med det anvendte antibiotikum azithromycin kan være forbundet med følgende bivirkninger i hht. www.medicin.dk

Almindelige (1-10 %)	Kvalme, opkastning, abdominalsmerter, diarré, forhøjede levertransaminaser.
Ikke almindelige (0,1-1 %)	Svimmelhed, sløvhed, kramper.<br>Hudkløe, hududslæt.<br>Candidiasis.<br>Vaginitis.
Sjældne (0,01-0,1 %)	Leverpåvirkning.<br>Sjældne kardiologiske, hæmatologiske og neurologiske bivirkninger.<br>Allergiske reaktioner, anafylaktisk reaktion.<br>Nyresvigt.<br>Høretab, tinnitus.

Forsøgspersoner anbefales straks at tage kontakt med den projektansvarlige læge, hvis forsøgspersonen får en bivirkning eller evt. får en bivirkning, som ikke er nævnt her.
Forsøgspersonen kan løbende i hele studieperioden (dag 0 – dag 30) tage kontakt til den projektansvarlige læge på telefon og e-mail.

Projektansvarlige læge Hans Linde Nielsen
Infektionsmedicinsk afdeling, Aalborg Sygehus
Tlf.: 99 32 65 32
e-mail: halin@rn.dk

Som led i projektet skal patienterne aflevere 3 spytprøver samt 2 ekstra afføringsprøve. Dette indebærer fraset ubehaget ingen risici for patienten. Patienten skal også afgive en blodprøve og i forbindelsen med dette kan der forekomme let ubehag, og der kan forekomme en lille blodansamling ved stik gennem blodåren. Desuden er der en minimal risiko for infektion i huden og lokal irritation ved prøvetagningsstedet.

Forsøgsdeltagerne informeres mundtligt og skriftligt i henhold til Helsinki deklarationen (5. udgave), og Den Centrale Videnskabsetiske Komité's skrivelse "Forsøgspersonens rettigheder i et biomedicinsk forskningsprojekt" udleveres, ligesom der udleveres skriftlig deltagerinformation.

Videnskabsetisk redegørelse
Diarré af enhver årsag er forbundet med betydelige gener for den enkelte patient, og betydelige økonomiske omkostninger for den enkelte og for samfundet i forbindelse med sygemeldinger og evt. undersøgelser og behandling. En nærmere belysning af årsager til infektiøs diarre kan føre til bedre

forebyggelse og behandling. Forsøgsdeltagerne informeres mundtligt og skriftligt i henhold til Helsinki deklarationen (5. udgave), og patienterne vil få udleveret Den Centrale Videnskabsetiske Komité's skrivelse: "Forsøgspersonens rettigheder i et biomedicinsk forskningsprojekt", ligesom der udleveres skriftlig deltagerinformation. Der indhentes informerende samtykke ved samtalen efter der har været tid til at læse det udleverede skriftlige materiale igennem. Patienten underskriver og daterer samtykkeerklæringen i to eksemplarer og beholder selv et eksemplar. Den projektansvarlige læge attesterer samtidigt, at mundtlig information har fundet sted og at skriftlig information er udleveret. Deltagelse i undersøgelsen er frivillig, og indhentede oplysninger vil blive behandlet strengt fortroligt. Deltagende personer kan til enhver tid vælge at trække sig ud af undersøgelsen, uden at det vil få konsekvenser for fremtidig behandling. Projektet anmeldes til Datatilsynet som forskningsbiobank og den del af fæcesprøverne (få ml) der ikke umiddelbart anvendes i projektet opbevares nedfrosset på Klinisk Mikrobiologisk Afd., Aalborg Sygehus med henblik på yderligere forskning i tilfælde af at nye metoder udvikles. Fæcesprøverne vil blive opbevaret og analyseret i Danmark, og vil ikke blive videreført til udlandet. De vil blive opbevaret i maximalt 5 år, hvorefter de vil blive destrueret. Nye projekter vil blive anmeldt til den regionale videnskabsetiske komite, og deltagerne anmodes på forhånd om tage stilling til, om de ønsker at få oplysning om resultater herfra, der kan have betydning for udvikling af sygdomme eller behandling. Formålet med denne undersøgelse er således at belyse den ætiologiske betydning af *C. concisus* ved diarré, og vurdere effekt af behandling af infektion med denne bakterie. På den baggrund er det nødvendigt at foretage en placebokontrolleret undersøgelse. Azithromycin er et præparat der rutinemæssigt anvendes til en række infektioner. Bivirkninger ved behandlingen kan forekomme, se ovenfor. Behandlingen kan muligvis gavne de patienter der efter lodtrækning modtager azithromycin behandling, og det kan komme fremtidige patienter til gavn. Hvis patienten fortsat har diarré efter 10 dages observationsperiode, tilbydes via telefoninterview, et andet besøg med den projektansvarlige læge og derefter behandling med "Cross-over"medicin. Dvs. hvis der initialt er givet placebo, behandles i anden omgang med azithromycin.
Det skønnes at opnåelsen af denne viden står i rimeligt forhold til de skitserede risici og ulemper patienterne udsættes for. Det skønnes ikke at projektet rummer en interessekonflikt mellem erhvervelse af ny viden og samfunds- eller økonomiske interesser.

Økonomiske aspekter
Projektet er en videreførelse af et afsluttet ph.d. projekt, og den projektansvarlige læge, vil blive aflønnet i forhold til dette. Supplerende finansiering vil blive søgt i nationale og internationale fonde. Patienter forventes at indgå uden økonomisk kompensation, men kan få transportudgifter dækket.

Deltagerinformation til forsøgspersonen

Campylobacter concisus placebokontrolleret behandlingsstudie

Vi henvender os til dig for at spørge, om du vil deltage i et videnskabeligt projekt. Deltagelse er frivilligt, så læs nedenstående grundigt og den generelle skrivelse ”Forsøgspersoners rettigheder i et biomedicinsk forskningsprojekt” før du beslutter dig. Du har ret til betænkningstid og til at medbringe en bisidder ved informationssamtalen.

Undersøgelsens baggrund
Mave- tarminfektioner er meget almindelige. Infektionerne overføres ved kontaktsmitte eller via levnedsmidler. Rutinemæssigt undersøges patienter for bakterierne Salmonella, Campylobacter og Yersinia. Herudover, kan andre bakterier, parasitter og virus forårsage mave- tarminfektioner.
En ny undersøgelse på Aalborg Sygehus, har vist at der findes en ny art af Campylobacter bakterien (benævnt, *Campylobacter concisus*), som forekommer næsten ligeså hyppigt som den almindelige form for Campylobacter. Symptombilledet for denne Campylobacter art er formentligt også opkastning, diarré og feber. Infektionen kan optræde alle aldre, men vores viden om, hvor bakterien kommer fra, og om patienterne kan have effekt af såkaldt antibiotisk behandling er meget begrænset.

Undersøgelsens formål
Formålet med undersøgelsen er derfor at undersøge om behandling med antibiotika af *Campylobacter concisus* bakterier kan give bedring af diarré episodens varighed og alvorlighed.

I din afføringsprøve fra den ___________(dato) har vi netop dyrket denne Campylobacter art, og vil spørge om du vil deltage i en undersøgelse af om antibiotisk behandling kan hjælpe på symptomerne.

Hvordan foregår undersøgelsen
Du vil blive behandlet med et antibiotikum (azithromycin) eller med placebo i form af en tablet dagligt i 3 dage. Inden opstart af behandling vil du blive bedt om at afgive en blodprøve til at måle det såkaldte infektionstal og væsketal. Ligeledes vil du blive bedt om at afgive en spytprøve. For at få oplysninger om din sygdomsepisode, vil vi bede dig om at udfylde et spørgeskema før opstart af behandling. De efterfølgende 10 dage skal du føre diarré-dagbog med daglige observationer i 10 dage. Spørgeskemaerne incl. Diarré-dagbog udleveres, og kan efterfølgende indsendes i de frankerede svarkuverter.
7 dage efter endt behandling vil du blive bedt om at indsende endnu en spytprøve samt en afføringsprøve. Afslutningsvis skal du 30 dage efter antibiotikum/placebo indsende endnu en spytprøve og afføringsprøve. Prøvetagningsvejledning er vedlagt og prøverør vil blive udleveret.
Spytprøver og afføringsprøverne bliver undersøgt for bakterier (inkl. Campylobacter concisus), parasitter og virus.
Efter 10 dage, med daglige observationer vil du på ny blive ringet op af den projektansvarlige læge med henblik på opfølgning mv. Hvis du fortsat har svære gener af din diarré, vil du blive tilbudt endnu en lægesamtale, hvor du tilbydes såkaldt ”Cross-over” medicin. Dvs. hvis du initialt fik placebo, får du anden gang antibiotikum (azithromycin). Herefter påbegyndes endnu en 10 dages periode med daglige observationer, med efterfølgende kontroldyrkning af spyt og afføring, samt en tredje telefonisk kontakt med den projektansvarlige læge.

Forsøgsdiagram ved dyrkning af *C. concisus*

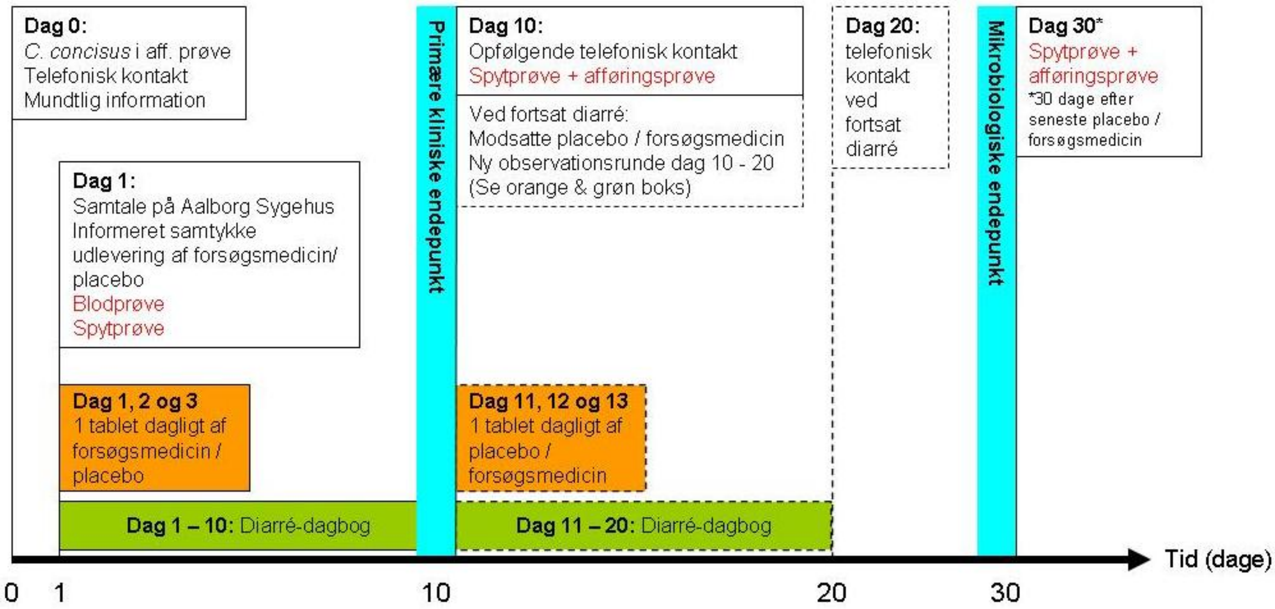

Studieperioden er fra 1. februar 2012 til 31. december 2013. Ved undersøgelsens afslutning vil resultaterne blive sammenlignet og offentliggjort, og du vil få skriftlig besked om undersøgelsens resultater. Yderligere vil du få skriftlig besked om hvilken forsøgsmedicin (azithromycin eller placebo) du fik i forsøget.

Undersøgelsen er godkendt som projekt af den Videnskabsetiske komité for Region Nordjylland, og projektet er i overensstemmelse med persondataloven og er anmeldt til Datatilsynet efter den procedure, der er aftalt mellem Datatilsynet og Region Nordjylland. Projektet omfatter 100 frivillige forsøgsdeltagere. Resultaterne offentliggøres i et lægevidenskabeligt eller andet tidsskrift med fuld anonymitet for dig. Herefter vil materialet med dine personlige data blive destrueret. Projektet anmeldes til Datatilsynet som forskningsbiobank og den del af afføringsprøverne (få ml) der ikke umiddelbart anvendes i projektet opbevares nedfrosset på Klinisk Mikrobiologisk Afd. på Aalborg Sygehus med henblik på yderligere forskning i tilfælde af at nye metoder udvikles. Afføringsprøverne vil blive opbevaret og analyseret i Danmark, og vil ikke blive videreført til udlandet. Nye projekter vil blive anmeldt til den regionale videnskabsetiske komite, og deltagerne anmodes på forhånd om tage stilling til, om de ønsker at få oplysning om resultater herfra, der kan have betydning for udvikling af sygdomme eller behandling.

Risici ved deltagelse
Som alle andre lægemidler kan azithromycin i anbefalede doser have bivirkninger. Se tabel 1.

Tabel 1. Behandling med det anvendte antibiotikum azithromycin kan være forbundet med følgende bivirkninger i hht. www.medicin.dk

Almindelige (1-10 %)	Kvalme, opkastning, abdominalsmerter, diarré, forhøjede leverenzymer.
Ikke almindelige (0,1-1 %)	Svimmelhed, sløvhed, kramper.<br>Hudkløe, hududslæt.<br>Svamp i munden.<br>Svamp i skeden.
Sjældne (0,01-0,1 %)	Blødning fra hud og slimhinder<br>Alvorlige overfølsomhedsreaktioner, f.eks. hævelser i huden, ansigt, læber, tunge, svælg eller hals.<br>Truende, evt. voldelig adfærd.<br>Kramper.<br>Uregelmæssig hurtig puls (hjerterytme).<br>Gulsot, leversvigt med sløjhed evt. bevidstløshed.<br>Hævelse af tunge, læber og ansigt.<br>Nyrebetændelse med smerter, feber og blod i urinen. Nyresvigt.<br>Betændelse i huden, især på hænder og fødder samt i og omkring munden ledsaget af feber.<br>Kraftig afskalning af huden.<br>Betændelse i bugspytkirtlen (voldsomme mavesmerter og feber).<br>Langvarig eller blodig diarré med mavesmerter eller feber, som kan være tegn på alvorlig tarmbetændelse (pseudomembranøs colitis)

Se: www.indlaegsseddel.dk under, tablet Zitromax (handelsnavn) for yderligere oplysninger om azithromycin.

Kontakt straks den projektansvarlige læge, hvis en bivirkning er generende eller bliver værre, eller hvis De får bivirkninger, som ikke er nævnt her.
De kan i hele studieperioden (dag 0 – dag 30) tage kontakt til den projektansvarlige læge på telefon og e-mail.

Projektansvarlige læge Hans Linde Nielsen, Infektionsmedicinsk afdeling, Aalborg Sygehus,
Tlf.: 99 32 65 32, e-mail: halin@rn.dk

Som led i projektet skal du aflevere 3 spytprøver samt 2 ekstra afføringsprøve. Dette indebærer fraset ubehaget ingen risici. Du skal også afgive en blodprøve og i forbindelsen med dette kan der forekomme let ubehag, og der kan forekomme en lille blodansamling ved stik gennem blodåren. Desuden er der en minimal risiko for infektion i huden og lokal irritation ved prøvetagningsstedet.
Svar på blodprøven vil blive set af den projektansvarlige læge, og du får ikke umiddelbar information herpå, med mindre der vises voldsomme forhøjede/skæve værdier, der kræver yderligere behandlingstiltag af den projektansvarlige læge.

Nulig gavnlig virkning
Behandlingen kan muligvis gavne de patienter der efter lodtrækning modtager antibiotika (azithromycin), og det kan komme fremtidige patienter til gavn. Det skønnes at opnåelsen af denne viden står i rimeligt forhold til de skitserede risici og ulemper. Projektdeltagelse skal ikke, ved vedvarende diarré, hindre evt. yderligere undersøgelse og behandling hos din vanlige læge.

Udelukkelse undervejs i projektet vil ske i tilfælde af:

- at du har allergi overfor azithromycin eller placebo, eller
- du bliver gravid under undersøgelsen.

Økonomi
Projektet er en videreførelse af et afsluttet ph.d. projekt, og den projektansvarlige læge, vil blive aflønnet i forhold til dette. Projektet er finansieret via fondsmidler fra Det Obelske Familiefond, se www.obel.com
Patienter forventes at indgå uden økonomisk kompensation, men kan få transportudgifter dækket.

Ved behov for yderligere information kan nedenstående projektansvarlige læge kontaktes

Med venlig hilsen

Hans Linde Nielsen
Projektansvarlig Læge
Infektionsmedicinsk Afdeling
Aalborg Universitetshospital
Mølleparkvej 4
9000 Aalborg
Telefon 99 32 65 32
Email: halin@rn.dk

Henrik Nielsen
Professor, ledende overlæge, dr med
Infektionsmedicinsk Afdeling
Aalborg Universitetshospital
Mølleparkvej 4
9000 Aalborg

Prøvetagningsvejledning

Spytprøve:
I prøveglasset kommes ca. 1 ml. spyt, lægges i filtpapiret og lægges tilbage i kuverten. Sendes med almindelig post, og portoen er betalt.

Afføringsprøve:
Fyld højst prøveglasset med 1/3 Fæces.

Prøvemængde:
Ca. 5 - 10 ml fæces er tilstrækkeligt.

Prøvetagning:
Frisk kvitteret fæces. Opsamling kan ske i wc-kumme præpareret med rigeligt toiletpapir.
Fæces kommes vha. den hvide prøvetagningsske i prøveglasset, lukkes tæt og kommes i det grå yderhylster.
Prøven er herefter klar til forsendelse i kuverten.

Opbevaring
I køleskab (5°C).

Forsendelse:
Almindelig post. Forsendes med postvæsenet i portofrit emballage.
transporttiden bør være højst 24 timer.

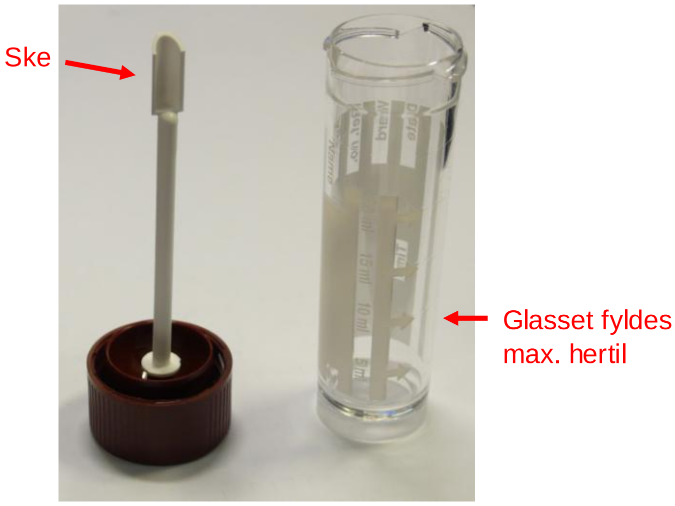

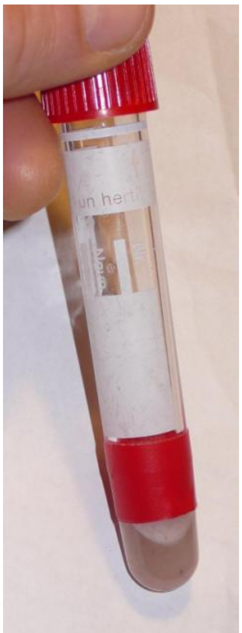
Spytprøve

Læge, Hans Linde Nielsen, Telefon: 99 32 65 32, E-mail: halin@rn.dk
