# Supplementary material for: Azithromycin vs. Placebo for the Clinical Outcome in Campylobacter concisus Diarrhoea in Adults: A Randomized, Double-Blinded, Placebo-Controlled Clinical Trial
Source: PLoS One. 2016 Nov 28;11(11):e0166395. doi: 10.1371/journal.pone.0166395 (PMC5125586; doi:10.1371/journal.pone.0166395)
Supplement: S2 File — (PDF) [file pone.0166395.s002.pdf]

Campylobacter concisus and diarrhea

A phase 3, single-centre, randomized, double-blinded, placebo-controlled study comparing the efficacy of 500 mg once-daily dose of azithromycin with a 500 mg once-daily dose of placebo for three days, for the treatment of *C. concisus* diarrhea in adult patients

Sponsorinvestigator:

Hans Linde Nielsen, M.D., ph.d.
Dpt. Infectious Diseases,
Aalborg University Hospital
Mølleparkvej 4, Postboks 365
9000 Aalborg
Telefone: +45 99 32 65 32
e-mail: halin@rn.dk

Sub-investigators:

Henrik Nielsen, Professor, DMSc.
Dpt. Infectious Diseases,
Aalborg University Hospital
Mølleparkvej 4, Postboks 365
9000 Aalborg
Telefone: +45 99 32 65 10
e-mail: Henrik.nielsen@rn.dk

Karina Frahm Kirk, M.D
Dpt. Infectious Diseases
Aalborg University Hospital
Mølleparkvej 4, Postboks 365
9000 Aalborg
e-mail: kfk@rn.dk

Jacob Bodilsen, M.D.
Dpt. Infectious Diseases
Aalborg University Hospital
Mølleparkvej 4, Postboks 365
9000 Aalborg
Telefone: +45 99 32 65 35
e-mail: jacob.bodilsen@rn.dk

Monitor:

GCP-enheden ved Aarhus Universitets Hospital
Olof Palmes Allé 15
8200 Aarhus N
Telefon: 99 32 62 44

Other partners and Scientific staff:

Marie Nørgaard, quality coordinator
Dpt. Of Clinical Biochemistry.
Aalborg University Hospital
Hobrovej 18-22
9000 Aalborg

Tove Ejlertsen, M.D., ph.d.
Dept. Of Clinical Microbiology.
Aalborg University Hospital
Mølleparkvej 8 A
9000 Aalborg
Telefone: +45 99 32 32 07
e-mail: Tove.ejlertsen@rn.dk

Glostrup Pharmacy, att. Pharmacist Steen Mejlgaard
Hovedvejen 101
2600 Glostrup
Telefone: +45 43 96 00 20
Fax: +45 43 96 07 67
e-mail: glostrup@apotekeren.dk
www.glostrup-apotek.dk

Background

Bacterial diarrhea

In the general population there is a high incidence of diarrheal illness primarily acquired through ingestion of contaminated food. *Campylobacter jejuni* is the leading cause of bacterial diarrhea in the Western world [1,2]. The family of *Campylobacteracea*e currently includes 21 species, which is isolated from humans (www.bacterio.cict.fr). They constitute, inter alia, *C. jejuni, C. coli, C. concisus, C. upsaliensis, C. lari, C. curvus, C. rectus, C. showae, C. gracilis, C. hominis, C. sputorum, C. fetus, C.* and *C. hyointestinalis lanienae. C. jejuni is* considered to be the leading cause of *Campylobacter*enteritis. *C. jejuni* can be found as part of the normal intestinal flora in a wide variety of animals, mainly birds. The infection occurs most often through contaminated food, mostly during industrial processing of poultry [3]. Diagnosis is achieved by culturing the bacteria from feces and in severe cases by culturing the bacteria from blood. Stool samples from general practice and hospital departments submitted to the clinical microbiology departments examines routinely only for *C. jejuni* and *C. coli* [4]. The split between *C. jejuni* and *C. coli* is estimated at approximately 95% and 5%, and clinical distinction is made between *C. jejuni* and *C. coli* [5].

Campylobacter jejuni

In 2009 in Denmark registered 3352 cases of *C. jejuni* infections [6]. The number of registered cases represents only the tip of the iceberg and it is assumed that many more people with bacterial diarrhea, have so mild disease manifestations that they have not sought medical attention. The department of Clinical Microbiology at Aalborg University Hospital performs bacteriological tests for North Jutland. In 2009, the department conducted cultivation of 16,035 stool samples from 5,947 patients and found 337 patients with *C. jejuni*. Three consecutive stool samples are suggested, to achieve a greater possibility of a positive finding [7]. Local data from the Department of Clinical Microbiology, Aalborg University Hospital shows that a bacteriological cause of the diarrhea that there can be cultured is only found in only approximately 12% of all bacterial stool samples. The remaining 88% are due to other causes such as viruses, parasites and not identifiable bacteria – but also many non-infectious causes such as inflammatory bowel disease, side effects to medication, diverticulitis and malabsorption e.i. Enteritis with *C. jejuni* is most common among children and young adults, but can occur any ages [8].

Campylobacter jejuni infection

C. jejuni first colonizes the small, than the large intestine, invading the intestinal mucosa with concurrent dysfunction, resulting in diarrhea. The bacteria can, but rarely does cross into the bloodstream and cause an invasive infection [9]. After an incubation period of 1-3 days the disease begins with general malaise, fever and flu-like symptoms. Diarrhea can be bloody, accompanied by cramping and abdominal pain. The disease is almost always self-limiting and extends from a few days and up to 1-2 weeks [10]. Enteritis with *C. jejuni* thus presents as aa spectrum from mild cases to severe illness, which may require hospitalization. The treatment is fluid therapy and antibiotics for susceptibility testing, usually with macrolide antibiotic [11]. After infection with *C. jejuni*, it is well known that 10 to 30% of patients experience persistent genes from their bowels, such as irritable bowel syndrome [12-15]. This disease is characterized by altered bowel habits and intermittent abdominal pain over at least 3 months without any detectable organic lesions, according to the so-called Rome criteria [16]. Infection by *C. jejuni* can also provide extraintestinal manifestations weeks or months after the primary diarrhea episode, for example in the form of reactive arthritis, which is seen in about 10-15% of cases of illness [17]. Guillain-Barré polyneuropathy is a rare, but serious complication. The disease is often preceded by

an infection and *C. jejuni* was for the first time in 1982 associated with this disease, and is today the most common triggering cause of the disease [18,19].

Prelavence of *C. concisus*

For *Campylobacter* species other than *C. jejuni* and C.coli, the frequency of, and the ability to induce diarrhea as well as the prevalence of complications are not as well documented. In the regular routine culturing a selective culture medium is used for *C. jejuni*, but it is well known that this method can not grow other *Campylobacter* species, including *C. concisus*. Here stool specimen are cultured using the so-called filter method in a microaerophilic gas mixture with added hydrogen [20]. For final identification of a possible *C. concisus* isolate molecular diagnostics is used, such as a specific real-time PCR based on the cpn60 gene [21].
A high incidence of *C. concisus* among children was already described in 1995 by a Lindblom. al [22]. In a large South African study, using the filter method as part of routine cultivation at a children's hospital in Cape Town, found 4,122 positve samples with *Campylobacter* species out of 19,535 stool samples, and *C. concisus* accounted for almost a quarter of the cultured *Campylobacter* species [23]. In a Danish study in 2002 Aabenhus used the filter method on 11,500 stool samples, and *C. concisus* was the most frequent isolated *Campylobacte r*species with 110 out of a total of 224 isolates [24].
In an unpublished study from Aalborg Hospital, stool samples sent to the species or serotype study at the department of Clinical Microbiology were cultivated for *C. concisus* using the filter method. In the course of 22 months, 10,388 stool samples were cultured for *C. concisus*, and grown in the usual way for other pathogenic intestinal bacteria such as *C. jejuni* and *Salmonella* species mm. The result was that there could be grown *C. concisus* from 378 patients with diarrhea. This was only surpassed by *C. jejuni*, with 456 patients in total. Patients with *C. concisus* were mainly young children and the elderly. Two thirds of the patients were from general practice, the remaining was from one of the region's hospitals. In about 10% cultivation of *C. concisus* was accompanied by a familiar gastrointestinal pathogen such as *Salmonella* species. Still, there is no clarity on whether *C. concisus* is as pathogen, and whether it can provide the same illness as *C. jejuni*, there is a questionnaire survey underway to elucidate this.
The source of infection and the reservoir for *C. concisus* is still undecided. In an Australian study, *C. concisus* grew in saliva samples in a very high number of patients and healthy carriers [25]. It is still undecided if the human oral cavity or gastrointestinal tract is the reservoir for *C. concisus*, and it is unknown whether healthy oral cavity-carriers are at greater risk of becoming ill with diarrhea.
There is still no knowledge of whether *C. concisus* requires treatment and whether possible antibiotic treatment can reduce the disease course in patients with diarrhea when the only culture findings in stool sample is *C. concisus*. A placebo-controlled study of the treatment effect by *C. concisus* culture-positive diarrhea could provide insights into the pathogenicity of *C. concisus* in our part of the world. It would also shed light on the need to diagnose a *C. concisus* infection.

Aim

The aim of this study is to investigate the effect of the antibiotic treatment with azithromycin in patients with diarrhea in which *C. concisus* isolated from the patient's stool sample as the only microorganism. There must not be another known gastrointestinal pathogenic microorganism as competing cause of the patient's symptoms. The effect is determined from questionnaires that illuminate the patient's general health and the development of diarrheal disease.
In parallel, cultivation of *C. concisus* from saliva and feces is carried out before and after treatment, to shed light on whether the eradication of *C. concisus* is possible by treatment with azithromycin.

Hypothesis

- Azithromycin is superior to placebo as antibiotic treatment for diarrhea caused by *C. concisus* and may shorten the course of illness.

- Azithromycin is superior to placebo eradicate *C. concisus* in saliva and stool.

Methods

Study Design
- Randomized, double-blind, placebo-controlled, parallel group clinical study of treatment effect by *C. concisus* culture-positive diarrhea.
- Azithromycin / placebo will be administered with one tablet of 500 mg daily for 3 days.
- Before treatment patients provide a stool sample.
- A diary regarding symptoms is kept from start of treatment to 7 days after cessation of treatment for a total observation period will be a total of 10 days.
- A stool sample and saliva sample are collected 7 days after cessation of treatment, and again after 30 days.
- If the participants have continuing diarrhea symptoms 10 days after the study, they are offered a new clinical assessment, as well as "cross-over" medication. A new observation period of 10 days is started after which the patient resubmits the stool and saliva samples.

Endpoints
The end point is the "change in the number of daily bowel movements", and disease duration.
The effect is measured by questionnaires including "diarrhea diary".

The primary endpoint:

- Number of days to achieve cessation of symptoms = "normal stool frequency", interpreted as <3 stools / day.

Secondary endpoints are:

- The number of daily bowel movements until achieving cessation of symptoms.

- The occurrence of secondary symptoms such as abdominal pain, nausea and vomiting

- Occurrence of adverse events compared to the placebo group.

- Eradication of *C. concisus* from saliva and feces.

Azithromycin
Azithromycin is an antibiotic from the macrolide group, which is mostly non-toxic. As previously mentioned, *C. jejuni* diarrhea is usually self-limiting, but treatment with a macrolide may be indicated in severe cases. There are several different kinds of macrolide antibiotics, and although there is not much scientific evidence for this, azithromycin is usually the first choice in the US and Europe [26-33]. (Http://www.cdc.gov/nczved/divisions/dfbmd/diseases/campylobacter/).
Azithromycin also has the advantage that it can be administered with only one tablet per day, which may increase the patient compliance. Resistance to antibiotics in the quinolone family is widespread and

widely used for empirical treatment of severe diarrhea incl. travelers' diarrhea [34]. The results from the cultivation of *C. concisus* in stool samples from the Clinical Microbiology Department at Aalborg Hospital also show that *C. concisus* up to now has been found sensitive to azithromycin. It has also been found that about one sixth of all *C. concisus* isolates are resistant to antibiotics in the quinolone family, why azithromycin should be regarded as the best choice of study antibiotic.

Farmacokinetics of azithromycin

Please see www.pro.medicin.dk

Azithromycin is acid stable. After oral intake, bioavailability is around 35%. After administration of 500 mg, peak plasma concentration is 0.4 micrograms / ml in 2-3 hours. Azithromycin breaks down quickly in the body. It penetrates the cells and accumulate intracellularly, especially in leucocytes and macrophages, where concentrations can be up to 100 times the plasma concentration. From these cells, the drug is secreted slowly to the interstitial space. The volume of distribution is large (20-30 l/kg). With multiple dose administration of 500 mg. a day for 3-5 days, maximum plasma concentration increase only to 0.6 micrograms/ml and does not pass the blood-brain barrier. Terminal plasma half-life is approximately 40 hours. Approximately 50% is excreted unchanged in the bile and about 12% through the kidneys.

Randomazation

Each patient is assigned a participant number and randomized to the study drug / placebo in a ratio of 1: 1. Pharmacist Steen Mejlgaard of Glostrup Pharmacy, Denmark, is responsible for labeling and packing of study medication (www.glostrup-apotek.dk). The randomization code is stored at the test center (in the Trial Master File) and will be broken only at study termination, unless by special circumstances, such as suspected serious adverse reactions, making code-breakage required. In need of information about each randomized patient during the project, Glostrup pharmacy can be contacted via telephone 43 96 00 20, which is open around the clock. Both the patient and the responsible physician is unaware of which treatment group each patient is randomized to. For each participant number is packed "Cross-over" medicine, for use if symptoms persist after 10 days.

Time period

The trial will be conducted from **1. april 2012** til **31. november 2013**.

Only patients over 18 years can participate. Patients must be residents of North Jutland. Patients are included among patients who are positive for *C. concisus* is a stool sample, while no other gatrointestinal microorganism is identified.

In the study period, all stool samples from adult patients were cultivated for *C. concisus,* patients from both general practice and from one of the hospitals in the North Denmark Region.

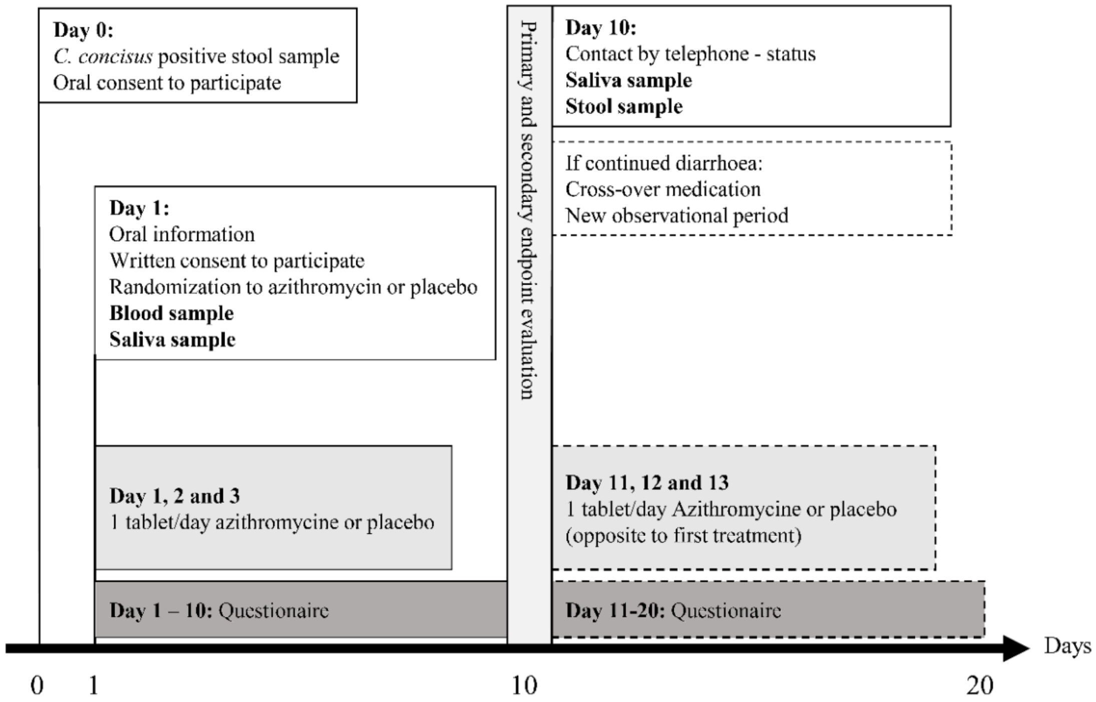

• Information for requesting / attending physician
Before the inclusion of the subject in the experiment, there has to be a relevant medical reason for obtaining a microbiological examination of the patient's stool. The patient's family doctor or a hospital doctor who ordered the study, is informed verbally about the trial by telephone by either sponsor investigator or a subinvestigator. The oral message will be followed by a written message to the ordering physician. General practitioners are informed via a letter from consultant practitioner Jan Nybo.

• First contact with the patient
After oral and written information is provided to the requesting / attending physician, and this person gives verbal consent to do so, the patient will be contacted by telephone by the sponsor investigator or a subinvestigator. A thorough oral information on the cultivation found by *C. concisus* in the patient's stool sample is given. If the patient still has diarrhea and no other pathogenic microorganism is identified, the patient will be invited to an interview with the sponsor investigator. There will also be informed of the number of days that the project extends over.

• 1st clinical visit between the patient and the person responsible physician.
The interview will take place in undisturbed surroundings in an interview room in the infectious diseases department at Aalborg Hospital. The patient will receive thorough oral and written information about the study, and will be accounted for side effects from the medication mm. Patients will receive:
• The National Research Ethics Committee's letter: "The subject's rights in a biomedical research."
• Written information for participants.
• Stool Sampling Guide.
• Copies for their own use of informed consent.
• Copies for their own use of power of attorney.

Informed consent is obtained after there has been time to read the issued written material through. The patient has the right to think participation over, and to bring a confidant to the interview. The

patient signs and dates both the consent form and proxy statement for each duplicate and will retain a copy of each. The project responsible physician certifies simultaneously that oral information has taken place and that the written information has been given. If the patient to provide written consent, the patient will be treated for 3 days with the tablet, azithomycin, 500mg, or placebo, with one tablet a day for 3 days. The placebo will be in a tablet of the same appearance, size and odor as a tablet containing azithromycin.

Labels for both azithromycin and placebo be written in Danish and will include:
1- “For clinical trials”
2- The contents are tablets and the number of tablets in the containers.
3- Code Name - in the form of student number.
4 Batch number - Indication that allows maintenance of blinding
5- Manufacturer
6- Expiration
7- Dosage: 1 x 1 tablet daily for 3 days
8- The investigator name: Hans Linde Nielsen, Department of Infectious Diseases, Aalborg Hospital, Tel .: 99 32 65 32 and email: halin@rn.dk

The patient is informed that there must not be taken "quenching" nanomedicine in the form of Loperamide, during the study period.

At the interview, the patient will be informed about the completion of a total of 2 questionnaires:
1- The first questionnaire filled out before starting treatment. It concerns the patient's general health information, and the date and symptoms of diarrheal disease.
2- The second questionnaire to be filled continuously for the next 10 days. The patient must keep a daily diary of diarrheal symptoms, and thus the development of this. That is, the patient must record progress during and after the 3-day treatment.

In the interview the patient will be asked to give a saliva sample. In addition, the patient will be asked to submit a saliva sample and stool sample 10 days after the first visit, for which handling utensils and a sampling manual are provided.

On the first visit, the patient will provide a blood test to measure infection counts.

Through completion of the questionnaire information on patients' clinical course are obtained and possible side effects to the treatment can be evaluated.

10 days after treatment, the patient is contacted by phone again by the sponsor investigator for a telephone interview on monitoring of side effects, and the patient's illness. If the patient 10 days after treatment remains bothered by diarrhea, a second visit is offered and "Cross-over" medicine, with subsequent observation period of 10 days can be initiated. If the patient initially received a placebo, treatment with azithromycin is started after 10 days. Likewise, if the patient initially has received azithromycin the patient is treated with placebo for 10 days. "Cross-over" medicine is also blinded to both the investigator and the subjects.
After the "cross-over" medicine, and subsequent observation period for another 10 days another telephone interview is conducted after 10 days. If symptoms of diarrheal disease continue 20 days after the study, the project responsible physician assesses if further treatment measures off-protocol should be taken, by referral to a specialized department or the patients GP.

The patient submit another stool sample and saliva sample after the 20 day observation period. Finally, the patient submits a final stool sample and saliva sample 30 days after the first visit. If the patient has received "Cross-over" drugs, samples must be submitted 30 days thereafter.

Compliance
The subject should consume 100% of the study medication during the study period. Compliance assessed partly by interview and partly by counting handed and returned study medication.

Side effects, risks and disadvantages
Please see http://pro.medicin.dk/Medicin/Praeparater/1745 for more information. Antibiotics of the macrolide azithromycin group are overall non-toxic. The most common side effects are anorexia, nausea, abdominal pain and transient diarrhea (See table below). Azithromycin is the antibiotic in the macrolide group has the best safety profile.

Side effects and events that take place during the trial record of the patient handed diary and reviewed with the project manager doctor who also sponsor investigator on the project. If any side effects become intolerable or poses a health risk to the patient immediately withdrawn from the study and appropriate measures will be taken for further monitoring and treatment.

Reports to the Danish Medicines Agency
The Sponsor Investigator will promptly notify the Danish Medicines Agency if unexpected serious suspected adverse reactions (Suspected Unexpected Serious Adverse Reactions, SUSARs) occur. See Medicines Act § 89 paragraph. 2. no. 1st
The definitions are as follows:
• Event: any adverse event in a patient or a subject in a clinical trial after treatment with a drug, without there necessarily being a relationship between this treatment and the adverse event
• Adverse Reaction: All unintended responses to an investigational compound at any dose.
•Unexpected adverse reaction means an adverse reaction, where the nature or severity is not consistent with the product information (eg investigator-Brochure for an unapproved investigational drug or product characteristics, if there is an aproved product)
• Serious adverse event is an event or effect that at any dose results in death, is life-threatening, requires hospitalization or prolongation of existing hospitalization, results in persistent or significant disability or incapacity, or is a congenital anomaly or birth defect.

The Sponsor Investigator records and reports all information about SUSARs that are fatal or life-threatening, to the Danish Medicines Agency as soon as possible and no later than 7 days after the sponsor investigator becomes aware of such possible side effects.

The Sponsor Investigator will, within 8 days, notify the Medicines Agency of all relevant information on Sponsors investigators follow-up alert.

All other SUSARs will be reported by the sponsor investigator to the Danish Medicines Agency within 15 days after the sponsor investigator became aware of these.

Reporting will be in drug Agency's website at the following link:

http://laegemiddelstyrelsen.dk/da/topics/bivirkninger-og-forsoeg/bivirkninger/meld-en-bivirkning-eller-utilsigtet-haendelse/kliniske-forsoeg/indberetning-af-mistaenkte-uventede-og-a---e-blanket

The following summary of product characteristics will be applied when assessing whether a serious related adverse reaction (SAR) is unexpected / expected and thus possibly becomes a SUSAR.

http://www.produktresume.dk/docushare/dsweb/Get/Document-27937/Azithromycin+2care4+%282care4%29%2C+filmovertrukne+tabletter+500+mg.doc

Guideline from the EU will also be followed
(Detailed guidance on the collection, verification and presentation of adverse event / reaction reports Arising from clinical trials on medicinal products for human use ('CT-3'))

Direct link:
http://ec.europa.eu/health/files/eudralex/vol-10/2011_c172_01/2011_c172_01_en.pdf

The subjects that complete day 10 (possibly Day 20 if "Cross-over) will speak to the sponsor investigator by telephone conversation. Sponsor Investigator is however available for subjects throughout the study period from day 0 to day 30 (last stool sample), and can be contacted regularly by phone and e-mail.

Study subjects
100 patients should be enrolled with 50 patients in the placebo group and 50 patients in the treatment group. Patients who are estimated to drop out of the study during the trial is set to 10% in each group. The time frame could be extended to reach a high enough number of participants.

Number of patients for study entry is calculated based on the following assumptions.
Type 1 errors, 2a = 0.05
Type 2 error, b = 0.20
Expected effect of placebo: 0.20
Expected effect of azithromycin: 0.60
Statistical power: 80

The number is calculated using. Program: STATA using. Command "db sampsi".
Assumptions:
- Average duration of diarrhea in the placebo group = 7
- Standard Deviation in the placebo group = 5
- Mean duration of diarrhea in the group azithromycin = 4
- Standard Deviation in the azithromycin group = 5

- N (placebo) / n (azithromycin) = 1
n (placebo) = 44
n (azithromycin) = 44

Inclusion criteria
• Diarrhea patients over 18 years of positive *C. concisus* culture from stool.

• Continued diarrhea defined as 3 or more watery to mushy stools daily
or
• 2 water thin to mushy stools, as well as at least one or more accompanying symptoms, such as abdominal pain, nausea, vomiting or fever.
• Symptoms for a minimum of 24 hours.
• Onset of symptoms of a maximum of 21 days.
• Oral and written consent and provide evidence that all relevant information about the study is given to the patient.
• The patient must be willing and able to appear for the scheduled visit and observe the planned curriculum.

Exclusion criteria
• Hypersensitivity to azithromycin, erythromycin, macrolide or ketolide antibiotic, or to any of the excipients.
• Age <18
• Pregnancy or lactation.
• Positive stool sample for other gastrointestinal microorganisms.
• Treatment with other antibiotics (at any time from 14 days before the first stool sample).
• Patients with colostomy and ileostomy.
• Patients with severe liver disease.
• Patients with severe renal impairment (GFR <10 ml / min).
• Patients with congenital or documented acquired QT prolongation.
• Patients treated with other active substances that prolong QT interval such as antiarrhythmics of classes IA and III, cisapride and terfenadine.
• Patients with electrolyte imbalance, particularly hypokalemia and hypomagnesemia.
• Patients with clinically relevant bradycardia, cardiac arrhythmia or severe heart failure.
• Chronic inflammatory bowel disease.
• Chronic diarrhea of another identifiable cause.
• Dementia.
• Serious illness less than 21 days from the planned entry into the study.
• Treatment with medication that has interaction with azithromycin for example alkaloids, cyclosporin or amiodarone.
• Patients involved in the planning or execution of the study.

Relevant pathogenic intestinal bacteria, viruses and parasites
Bacteria: *Salmonella, Shigella, Yersinia*, *Campylobacter jejuni, Aeromonas, Plesiomonas, Vibrio, Clostridium difficile, E. coli* (EHEC, VTEC) EPEC, A / EEC, EAggEC, EIEC) Viruses: Norovirus, Rotavirus, enteric adenovirus, Parasites : *Giardia lamblia* and *Cryptosporidium parvum.*

The feces are stored for both virus and parasite survey of Clinical Microbiology Department. Aalborg Hospital.
• App. 200 mg faeces s frozen in beef broth with glycerol 10% by -80C for later use.
The tube marked with number and freezer allocation on the lid and put in the freezer box (SSI item no. 75699) labeled with "Feces: *C. concisus* & Azithromycin". The samples are placed in the project freezer space at the Clinical Microbiology Department. Aalborg Hospital.

• App. 200 mg are stored in empty 2ml eppendorf tubes (SSI No. 5998) (-20C for later virus and parasite studies). The samples were placed in the project freezer space at Clinical Microbiology Department. Aalborg Hospital.

Data management
Information on the subject is protected under the Act on processing personal data and health law, and authorization for the use of patient-related data (medical records from hospitalized patients and laboratory results) is obtained before use. Data collection and handling will take place after the GCP's guidelines, like the study conducted by CONSORT recommendations.
http://www.consort-statement.org/
The trial will be pre-authorized and monitored by the GCP unit at Aarhus University.
http://gcp.au.dk/
The trial will also be registered in the US database:
http://www.clinicaltrials.gov/

Economic aspects
The project is a continuation of a completed Ph.D. project, and the project leader doctor continues his current research position and will be paid in relation to this. Additional funding will be sought in national and international funds. Patients included without financial compensation, but can get travel expenses covered.

Ethical aspects
Diarrhea is associated with significant inconvenience to the individual patient, and have significant economic costs for the individual and also for society in sickness and work leave, health examinations and treatment. A clarification of the causes of infectious diarrhea can lead to better prevention and treatment. Patients can be treated with a common antibiotics (azithromycin), which has been used for many years in Denmark, inter alia, for the treatment of Chlamydia.
The purpose of this study is to elucidate the etiologic role of *C. concisus* as a cause of diarrhea and assess the effect of treatment of infection with this bacterium. Against this background, it is necessary to conduct a placebo-controlled study.
Azithromycin is a preparation routinely used in bacterial infections such as Chlamydia. Side effects of the treatment are few, but can occur, see table 1.
The study complies with the Helsinki Declaration (5th edition), and is submitted to the Research Ethics Committee of North Jutland and the Danish Medicines Agency. Moreover reported trial data monitoring and monitored by GCP unit at Aarhus University.
Participation in the survey is voluntary and the information gathered will be kept confidential, and the persons may at any time decide to withdraw from the study. Informed consent will be obtained from the patient. Stool samples (few ml) are not immediately used in the project are stored frozen in Clinical Microbiology Div., Aalborg Hospital with a possible view. Feces samples will be stored and analyzed in Denmark, and will not be sent abroad. They will be stored for a maximum of five years, after which they will be destroyed. New projects will be reported to the regional ethical committee, and participants requested in advance about to decide whether they want to get information about the results here, which may influence the development of disease or treatment. Sputum samples will not be frozen.
It is estimated that attainment of this knowledge is proportionate to the identified risks and benefits the patients are exposed to. As part of the project, patients must also deliver an extra stool sample. This means, apart from the discomfort, no risks to the patient. In connection with blood sampling may occur slight discomfort and there may be a small blood clot in plugs through the vein. Furthermore, there is a minimal risk of infection of the skin and irritation at the sampling site.

The questionnaire was validated by other studies of chronic bowel symptoms and general mental symptoms. It is estimated that the questionnaire may not be stressful to answer, or can cause permanent psychological damage. It is not expected that the project contains a conflict of interest between the acquisition of new knowledge and social or economic interests.

Publication

Results from the survey will be presented in national and international meetings and published in international peer-reviewed journals. Both positive and negative results will be published. All publication rights accrue the sponsor investigator. The Author order coincides with each individuals performance in relation to the publication.

Reference List

(1) Wingstrand A, Neimann J, Engberg J, Nielsen EM, Gerner-Smidt P, Wegener HC, et al. Fresh chicken as main risk factor for campylobacteriosis, Denmark. *Emerg Infect Dis* 2006; 12: 280-285.

(2) Zilbauer M, Dorrell N, Wren BW, Bajaj-Elliott M. *Campylobacter*jejuni-mediated disease pathogenesis: an update. *Trans R Soc Trop Med Hyg* 2008; 102: 123-129.

(3) Engberg J. Contributions to the epidemiology of *Campylobacter*infections. A review of clinical and microbiological studies. *Dan Med Bull* 2006; 53: 361-389.

(4) Andersen LP, Gerner-Smidt P, Jensen TE, Jensen KT, Lester A, Tvede M. Anbefalinger fra DSKM's arbejdsgruppe for tarmbakteriologi. 2003.
Ref Type: Generic

(5) Vandenberg O, Dediste A, Houf K, Ibekwem S, Souayah H, Cadranel S, et al. Arcobacter species in humans. *Emerg Infect Dis* 2004; 10: 1863-1867.

(6) S.Ethelberg, K.Mølbak, K.E.P.Olsen, F.Scheutz. Zoonotiske Tarminfektioner 2009. 2010.

(7) Ethelberg S, Olsen KE, Gerner-Smidt P, Molbak K. The significance of the number of submitted samples and patient-related factors for faecal bacterial diagnostics. *Clin Microbiol Infect* 2007; 13: 1095-1099.

(8) Engberg J. Contributions to the epidemiology of *Campylobacter*infections. A review of clinical and microbiological studies. *Dan Med Bull* 2006; 53: 361-389.

(9) Poly F, Guerry P. Pathogenesis of Campylobacter. *Curr Opin Gastroenterol* 2008; 24: 27-31.

(10) Zilbauer M, Dorrell N, Wren BW, Bajaj-Elliott M. *Campylobacter*jejuni-mediated disease pathogenesis: an update. *Trans R Soc Trop Med Hyg* 2008; 102: 123-129.

(11) Hansen NE, Haunsø S, Schaffalitzky De Muckadell OB. Medicinsk Kompendium, 16. udgave, 2004. 16. udgave ed. NYT NORDISK FORLAG ARNOLD BUSCK A/S 2004; 2004.

(12) Spiller RC. Role of infection in irritable bowel syndrome. *J Gastroenterol* 2007; 42 Suppl 17: 41-47.

(13) Talley NJ, Spiller R. Irritable bowel syndrome: a little understood organic bowel disease? *Lancet* 2002; 360: 555-564.

(14) Talley NJ. Irritable bowel syndrome. *Intern Med J* 2006; 36: 724-728.

(15) Spiller RC. Is IBS caused by infectious diarrhea? *Nat Clin Pract Gastroenterol Hepatol* 2007; 4: 642-643.

(16) Drossman DA. The functional gastrointestinal disorders and the Rome III process. *Gastroenterology* 2006; 130: 1377-1390.

(17) Townes JM, Deodhar AA, Laine ES, Smith K, Krug HE, Barkhuizen A, et al. Reactive arthritis following culture-confirmed infections with bacterial enteric pathogens in Minnesota and Oregon: a population-based study. *Ann Rheum Dis* 2008.

(18) Engberg JH. [Guillain-Barre syndrome and Campylobacter]. *Ugeskr Laeger* 2002; 164: 5905-5908.

(19) Hughes RA, Cornblath DR. Guillain-Barre syndrome. *Lancet* 2005; 366: 1653-1666.

(20) Engberg J, On SL, Harrington CS, Gerner-Smidt P. Prevalence of Campylobacter, Arcobacter, Helicobacter, and Sutterella spp. in human fecal samples as estimated by a reevaluation of isolation methods for Campylobacters. *J Clin Microbiol* 2000; 38: 286-291.

(21) Chaban B, Musil KM, Himsworth CG, Hill JE. Development of cpn60-based real-time quantitative PCR assays for the detection of 14 *Campylobacter*species and application to screening of canine fecal samples. *Appl Environ Microbiol* 2009; 75: 3055-3061.

(22) Lindblom GB, Sjogren E, Hansson-Westerberg J, Kaijser B. *Campylobacter*upsaliensis, C. sputorum sputorum and *C. concisus* as common causes of diarrhoea in Swedish children. *Scand J Infect Dis* 1995; 27: 187-188.

(23) Lastovica AJ. Emerging *Campylobacter*spp.: The tip of the iceberg. *Clinical Microbiology Newsletter - 1 April 2006 (Vol 28, Issue 7, Pages 49-56, DOI: 10 1016/j clinmicnews 2006 03 004)* 2010.

(24) Aabenhus R, Permin H, On SL, Andersen LP. Prevalence of *Campylobacter*concisus in diarrhoea of immunocompromised patients. *Scand J Infect Dis* 2002; 34: 248-252.

(25) Zhang L, Budiman V, Day AS, Mitchell H, Lemberg DA, Riordan SM, et al. Isolation and detection of *Campylobacter*concisus from saliva of healthy individuals and patients with inflammatory bowel disease. *J Clin Microbiol* 2010; 48: 2965-2967.

(26) Gendrel D, Cohen R. [Bacterial diarrheas and antibiotics: European recommendations]. *Arch Pediatr* 2008; 15 Suppl 2: S93-S96.

(27) Vukelic D, Trkulja V, Salkovic-Petrisic M. Single oral dose of azithromycin versus 5 days of oral erythromycin or no antibiotic in treatment of *Campylobacter*enterocolitis in children: a prospective randomized assessor-blind study. *J Pediatr Gastroenterol Nutr* 2010; 50: 404-410.

(28) Jones K, Felmingham D, Ridgway G. In vitro activity of azithromycin (CP-62,993), a novel macrolide, against enteric pathogens. *Drugs Exp Clin Res* 1988; 14: 613-615.

(29) Hill DR, Ericsson CD, Pearson RD, Keystone JS, Freedman DO, Kozarsky PE, et al. The practice of travel medicine: guidelines by the Infectious Diseases Society of America. *Clin Infect Dis* 2006; 43: 1499-1539.

(30) Hill DR, Beeching NJ. Travelers' diarrhea. *Curr Opin Infect Dis* 2010; 23: 481-487.

(31) Sanders JW, Frenck RW, Putnam SD, Riddle MS, Johnston JR, Ulukan S, et al. Azithromycin and loperamide are comparable to levofloxacin and loperamide for the treatment of traveler's diarrhea in United States military personnel in Turkey. *Clin Infect Dis* 2007; 45: 294-301.

(32) Adachi JA, Ericsson CD, Jiang ZD, DuPont MW, Martinez-Sandoval F, Knirsch C, et al. Azithromycin found to be comparable to levofloxacin for the treatment of US travelers with acute diarrhea acquired in Mexico. *Clin Infect Dis* 2003; 37: 1165-1171.

(33) Tribble DR, Sanders JW, Pang LW, Mason C, Pitarangsi C, Baqar S, et al. Traveler's diarrhea in Thailand: randomized, double-blind trial comparing single-dose and 3-day azithromycin-based regimens with a 3-day levofloxacin regimen. *Clin Infect Dis* 2007; 44: 338-346.

(34) Engberg J, Aarestrup FM, Taylor DE, Gerner-Smidt P, Nachamkin I. Quinolone and macrolide resistance in *Campylobacter*jejuni and *C. coli*: resistance mechanisms and trends in human isolates. *Emerg Infect Dis* 2001; 7: 24-34.
