# Supplementary material for: Azithromycin vs. Placebo for the Clinical Outcome in Campylobacter concisus Diarrhoea in Adults: A Randomized, Double-Blinded, Placebo-Controlled Clinical Trial
Source: PLoS One. 2016 Nov 28;11(11):e0166395. doi: 10.1371/journal.pone.0166395 (PMC5125586; doi:10.1371/journal.pone.0166395)
Supplement: S5 File — (PDF) [file pone.0166395.s005.pdf]

Spørgeskema dag 1 Dato (ddmmyy) ____________________

Er du blevet rask: Ja ☐, du behøver ikke at udfylde flere sider. Spørgeskemaet indsendes dag 10.
Nej ☐, udfyld venligst nedenstående og næste dag

Antal afføringer dags dato ___________________________ Skriv antal

Jeg har indtaget forsøgsmedicin dags dato ☐ (sæt kryds)

Har du haft: (Husk at afkrydse alle felter med enten nej eller ja)	Nej	Ja	Evt. kommentarer
Vandig afføring	☐	☐	________________
Slimet afføring	☐	☐	________________
Blodig afføring	☐	☐	________________
Natlig diarré	☐	☐	________________
Feber	☐	☐	Temperatur ______________ C°
Kulderysteler	☐	☐	________________
Kvalme	☐	☐	________________
Opkastning	☐	☐	Antal ________________
Hovedpine	☐	☐	________________
Svimmelhed	☐	☐	________________
Mavesmerter	☐	☐	________________
Muskelsmerter	☐	☐	________________
Vægttab	☐	☐	Hvis ja, din angiv vægt ______kg

Andre symptomer
____

Bivirkninger: ☐ Ja ☐ Nej ☐ Hvilken, hvilke
____
____

Spørgeskema dag 2 Dato (ddmmyy) ____________________

Er du blevet rask: Ja ☐, du behøver ikke at udfylde flere sider.
Spørgeskemaet indsendes dag 10.
Nej ☐, udfyld venligst nedenstående og næste dag

Antal afføringer dags dato ___________________________Skriv antal

Jeg har indtaget forsøgsmedicin dags dato ☐ (sæt kryds)

Har du haft: (Husk at afkrydse alle felter med enten nej eller ja)	Nej	Ja	Evt. kommentarer
Vandig afføring	☐	☐	___
Slimet afføring	☐	☐	___
Blodig afføring	☐	☐	___
Natlig diarré	☐	☐	___
Feber	☐	☐	Temperatur_______________C°
Kulderysteler	☐	☐	___
Kvalme	☐	☐	___
Opkastning	☐	☐	Antal___________________
Hovedpine	☐	☐	___
Svimmelhed	☐	☐	___
Mavesmerter	☐	☐	___
Muskelsmerter	☐	☐	___
Vægttab	☐	☐	Hvis ja, din angiv vægt ______kg

Andre symptomer
____

Bivirkninger: ☐ Ja ☐ Nej ☐ Hvilken, hvilke
____
____

Spørgeskema dag 3 Dato (ddmmyy) ____________________

Er du blevet rask: Ja □, du behøver ikke at udfylde flere sider.
Spørgeskemaet indsendes dag 10.
Nej □, udfyld venligst nedenstående og næste dag

Antal afføringer dags dato ___________________________Skriv antal

Jeg har indtaget forsøgsmedicin dags dato □ (sæt kryds)

Har du haft: (Husk at afkrydse alle felter med enten nej eller ja)	Nej	Ja	Evt. kommentarer
Vandig afføring	□	□	______________________________
Slimet afføring	□	□	______________________________
Blodig afføring	□	□	______________________________
Natlig diarré	□	□	______________________________
Feber	□	□	Temperatur _______________C°
Kulderysteler	□	□	______________________________
Kvalme	□	□	______________________________
Opkastning	□	□	Antal ________________________
Hovedpine	□	□	______________________________
Svimmelhed	□	□	______________________________
Mavesmerter	□	□	______________________________
Muskelsmerter	□	□	______________________________
Vægttab	□	□	Hvis ja, din angiv vægt ______kg

Andre symptomer
____

Bivirkninger: □ Ja □ Nej □ Hvilken, hvilke
____
____

Spørgeskema dag 4 Dato (ddmmyy) ____________________

Er du blevet rask: Ja □, du behøver ikke at udfylde flere sider. Spørgeskemaet indsendes dag 10.
Nej □, udfyld venligst nedenstående og næste dag

Antal afføringer dags dato ____________________________Skriv antal

Har du haft: (Husk at afkrydse alle felter med enten nej eller ja)	Nej	Ja	Evt. kommentarer
Vandig afføring	□	□	____________
Slimet afføring	□	□	____________
Blodig afføring	□	□	____________
Natlig diarré	□	□	____________
Feber	□	□	Temperatur_______________C°
Kulderysteler	□	□	____________
Kvalme	□	□	____________
Opkastning	□	□	Antal____________
Hovedpine	□	□	____________
Svimmelhed	□	□	____________
Mavesmerter	□	□	____________
Muskelsmerter	□	□	____________
Vægttab	□	□	Hvis ja, din angiv vægt ______kg

Andre symptomer

____

Bivirkninger: □ Ja □ Nej □ Hvilken, hvilke ____________________
____

Spørgeskema dag 5 Dato (ddmmyy) ____________________

Er du blevet rask: Ja ☐, du behøver ikke at udfylde flere sider.
Spørgeskemaet indsendes dag 10.
Nej ☐, udfyld venligst nedenstående og næste dag

Antal afføringer dags dato ____________________________Skriv antal

Har du haft: (Husk at afkrydse alle felter med enten nej eller ja)	Nej	Ja	Evt. kommentarer
Vandig afføring	☐	☐	________________
Slimet afføring	☐	☐	________________
Blodig afføring	☐	☐	________________
Natlig diarré	☐	☐	________________
Feber	☐	☐	Temperatur_______________C°
Kulderysteler	☐	☐	________________
Kvalme	☐	☐	________________
Opkastning	☐	☐	Antal________________
Hovedpine	☐	☐	________________
Svimmelhed	☐	☐	________________
Mavesmerter	☐	☐	________________
Muskelsmerter	☐	☐	________________
Vægttab	☐	☐	Hvis ja, din angiv vægt ______kg

Andre symptomer
____

Bivirkninger: ☐ Ja ☐ Nej ☐ Hvilken, hvilke
____
____

Spørgeskema dag 6 Dato (ddmmyy) ____________________

Er du blevet rask: Ja □, du behøver ikke at udfylde flere sider. Spørgeskemaet indsendes dag 10.

Nej □, udfyld venligst nedenstående og næste dag

Antal afføringer dags dato ____________________________Skriv antal

Har du haft: (Husk at afkrydse alle felter med enten nej eller ja)	Nej	Ja	Evt. kommentarer
Vandig afføring	□	□	______________________________
Slimet afføring	□	□	______________________________
Blodig afføring	□	□	______________________________
Natlig diarré	□	□	______________________________
Feber	□	□	Temperatur _______________C°
Kulderysteler	□	□	______________________________
Kvalme	□	□	______________________________
Opkastning	□	□	Antal___________________________
Hovedpine	□	□	______________________________
Svimmelhed	□	□	______________________________
Mavesmerter	□	□	______________________________
Muskelsmerter	□	□	______________________________
Vægttab	□	□	Hvis ja, din angiv vægt ______kg

Andre symptomer

____

Bivirkninger: □ Ja □ Nej □ Hvilken, hvilke

____

____

Spørgeskema dag 7 Dato (ddmmyy) ____________________

Er du blevet rask: Ja □, du behøver ikke at udfylde flere sider.
Spørgeskemaet indsendes dag 10.
Nej □, udfyld venligst nedenstående og næste dag

Antal afføringer dags dato ____________________________Skriv antal

Har du haft: (Husk at afkrydse alle felter med enten nej eller ja)	Nej	Ja	Evt. kommentarer
Vandig afføring	□	□	______________
Slimet afføring	□	□	______________
Blodig afføring	□	□	______________
Natlig diarré	□	□	______________
Feber	□	□	Temperatur_______________C°
Kulderysteler	□	□	______________
Kvalme	□	□	______________
Opkastning	□	□	Antal___________________________
Hovedpine	□	□	______________
Svimmelhed	□	□	______________
Mavesmerter	□	□	______________
Muskelsmerter	□	□	______________
Vægttab	□	□	Hvis ja, din angiv vægt ______kg

Andre symptomer
____

Bivirkninger: □ Ja □ Nej □ Hvilken, hvilke
____
____

Spørgeskema dag 8 Dato (ddmmyy) ____________________

Er du blevet rask: Ja □, du behøver ikke at udfylde flere sider.
Spørgeskemaet indsendes dag 10.
Nej □, udfyld venligst nedenstående og næste dag

Antal afføringer dags dato ____________________________Skriv antal

Har du haft: (Husk at afkrydse alle felter med enten nej eller ja)	Nej	Ja	Evt. kommentarer
Vandig afføring	□	□	____________
Slimet afføring	□	□	____________
Blodig afføring	□	□	____________
Natlig diarré	□	□	____________
Feber	□	□	Temperatur______________C°
Kulderysteler	□	□	____________
Kvalme	□	□	____________
Opkastning	□	□	Antal____________
Hovedpine	□	□	____________
Svimmelhed	□	□	____________
Mavesmerter	□	□	____________
Muskelsmerter	□	□	____________
Vægttab	□	□	Hvis ja, din angiv vægt ______kg

Andre symptomer
____

Bivirkninger: □ Ja □ Nej □ Hvilken, hvilke
____
____

Spørgeskema dag 9 Dato (ddmmyy) ____________________

Er du blevet rask: Ja □, du behøver ikke at udfylde flere sider.
Spørgeskemaet indsendes dag 10.
Nej □, udfyld venligst nedenstående og næste dag

Antal afføringer dags dato ____________________________Skriv antal

Har du haft: (Husk at afkrydse alle felter med enten nej eller ja)	Nej	Ja	Evt. kommentarer
Vandig afføring	□	□	________________
Slimet afføring	□	□	________________
Blodig afføring	□	□	________________
Natlig diarré	□	□	________________
Feber	□	□	Temperatur_______________C°
Kulderysteler	□	□	________________
Kvalme	□	□	________________
Opkastning	□	□	Antal_______________
Hovedpine	□	□	________________
Svimmelhed	□	□	________________
Mavesmerter	□	□	________________
Muskelsmerter	□	□	________________
Vægttab	□	□	Hvis ja, din angiv vægt ______kg

Andre symptomer
____

Bivirkninger: □ Ja □ Nej □ Hvilken, hvilke
____
____

Spørgeskema dag 10 Dato (ddmmyy) ____________________

Er du blevet rask: Ja □, du behøver ikke at udfylde flere sider.
Spørgeskemaet indsendes dag 10.
Nej □, udfyld venligst nedenstående og næste dag

Antal afføringer dags dato ____________________________Skriv antal

Har du haft: (Husk at afkrydse alle felter med enten nej eller ja)	Nej	Ja	Evt. kommentarer
Vandig afføring	□	□	____________________
Slimet afføring	□	□	____________________
Blodig afføring	□	□	____________________
Natlig diarré	□	□	____________________
Feber	□	□	Temperatur______________C°
Kulderysteler	□	□	____________________
Kvalme	□	□	____________________
Opkastning	□	□	Antal____________________
Hovedpine	□	□	____________________
Svimmelhed	□	□	____________________
Mavesmerter	□	□	____________________
Muskelsmerter	□	□	____________________
Vægttab	□	□	Hvis ja, din angiv vægt ______kg

Andre symptomer
____

Bivirkninger: □ Ja □ Nej □ Hvilken, hvilke
____
____
