# Supplementary material for: Azithromycin vs. Placebo for the Clinical Outcome in Campylobacter concisus Diarrhoea in Adults: A Randomized, Double-Blinded, Placebo-Controlled Clinical Trial
Source: PLoS One. 2016 Nov 28;11(11):e0166395. doi: 10.1371/journal.pone.0166395 (PMC5125586; doi:10.1371/journal.pone.0166395)
Supplement: S6 File — (PDF) [file pone.0166395.s006.pdf]

Questionnaire Day 1 Date (ddmmyy) ____________________

Are you cured: Yes □, You do not have to fill the next pages

No □, Please complete below and the following day

Number of stools a day ____________________________(numbers)

I have taken the study medication ☐ (tick)

Have you had:	No	Yes	Further comments
Watery stools	☐	☐	____________________
Mucus in stools	☐	☐	____________________
Blood in stools	☐	☐	____________________
Noctural diarrhoea	☐	☐	____________________
Fever	☐	☐	Temperatur_______________C°
Chills	☐	☐	____________________
Nausea	☐	☐	____________________
Vomiting	☐	☐	Numbers________________
Headache	☐	☐	____________________
Dizziness	☐	☐	____________________
Abdominal pain	☐	☐	____________________
Muscle aches	☐	☐	____________________
Weight loss	☐	☐	If yes, write weight _______kg

Other symptoms

____

Side effects ☐ Yes ☐ No ☐

Please explain:

____

____

Questionnaire Day 2 Date (ddmmyy) ____________________

Are you cured:	Yes	☐, You do not have to fill the next pages
	No	☐, Please complete below and the following day

Number of stools a day ____________________________(numbers)

I have taken the study medication ☐ (tick)

Have you had:	No	Yes	Further comments
Watery stools	☐	☐	______________________
Mucus in stools	☐	☐	______________________
Blood in stools	☐	☐	______________________
Noctural diarrhoea	☐	☐	______________________
Fever	☐	☐	Temperatur_______________C°
Chills	☐	☐	______________________
Nausea	☐	☐	______________________
Vomiting	☐	☐	Numbers________________
Headache	☐	☐	______________________
Dizziness	☐	☐	______________________
Abdominal pain	☐	☐	______________________
Muscle aches	☐	☐	______________________
Weight loss	☐	☐	If yes, write weight ______kg

Other symptoms

____

Side effects ☐ Yes ☐ No ☐

Please explain:

____

____

Questionnaire Day 3 Date (ddmmyy) ____________________

Are you cured:	Yes □, You do not have to fill the next pages		
	No □, Please complete below and the following day		
Number of stools a day ____________________________(numbers)			
I have taken the study medication ☐ (tick)			
Have you had:	No	Yes	Further comments
Watery stools	☐	☐	________________
Mucus in stools	☐	☐	________________
Blood in stools	☐	☐	________________
Noctural diarrhoea	☐	☐	________________
Fever	☐	☐	Temperatur______________C°
Chills	☐	☐	________________
Nausea	☐	☐	________________
Vomiting	☐	☐	Numbers________________
Headache	☐	☐	________________
Dizziness	☐	☐	________________
Abdominal pain	☐	☐	________________
Muscle aches	☐	☐	________________
Weight loss	☐	☐	If yes, write weight ______kg
Other symptoms ____________________________			
Side effects ☐ Yes	☐ No ☐		Please explain: ________________ ________________

Questionnaire Day 4 Date (ddmmyy) ____________________

Are you cured: Yes □, You do not have to fill the next pages

No □, Please complete below and the following day

Number of stools a day ____________________________(numbers)

Have you had:	No	Yes	Further comments
Watery stools	☐	☐	______________________________
Mucus in stools	☐	☐	______________________________
Blood in stools	☐	☐	______________________________
Noctural diarrhoea	☐	☐	______________________________
Fever	☐	☐	Temperatur_______________C°
Chills	☐	☐	______________________________
Nausea	☐	☐	______________________________
Vomiting	☐	☐	Numbers_______________________
Headache	☐	☐	______________________________
Dizziness	☐	☐	______________________________
Abdominal pain	☐	☐	______________________________
Muscle aches	☐	☐	______________________________
Weight loss	☐	☐	If yes, write weight ______kg

Other symptoms

____

Side effects ☐ Yes ☐ No ☐

Please explain:

____

____

Questionnaire Day 5 Date (ddmmyy) ____________________

Are you cured: Yes □, You do not have to fill the next pages

No □, Please complete below and the following day

Number of stools a day ____________________________(numbers)

Have you had:	No	Yes	Further comments
Watery stools	□	□	______________________
Mucus in stools	□	□	______________________
Blood in stools	□	□	______________________
Noctural diarrhoea	□	□	______________________
Fever	□	□	Temperatur_______________C°
Chills	□	□	______________________
Nausea	□	□	______________________
Vomiting	□	□	Numbers________________________
Headache	□	□	______________________
Dizziness	□	□	______________________
Abdominal pain	□	□	______________________
Muscle aches	□	□	______________________
Weight loss	□	□	If yes, write weight _______kg

Other symptoms

____

Side effects □ Yes □ No □ Please explain:

____

____

Questionnaire Day 6 Date (ddmmyy) ____________________

Are you cured: Yes □, You do not have to fill the next pages

No □, Please complete below and the following day

Number of stools a day ____________________________(numbers)

Have you had:	No	Yes	Further comments
Watery stools	☐	☐	______________________________
Mucus in stools	☐	☐	______________________________
Blood in stools	☐	☐	______________________________
Noctural diarrhoea	☐	☐	______________________________
Fever	☐	☐	Temperatur_______________C°
Chills	☐	☐	______________________________
Nausea	☐	☐	______________________________
Vomiting	☐	☐	Numbers_______________________
Headache	☐	☐	______________________________
Dizziness	☐	☐	______________________________
Abdominal pain	☐	☐	______________________________
Muscle aches	☐	☐	______________________________
Weight loss	☐	☐	If yes, write weight ______kg

Other symptoms

____

Side effects ☐ Yes ☐ No ☐ Please explain:

____

____

Questionnaire Day 7 Date (ddmmyy) ____________________

Are you cured: Yes □, You do not have to fill the next pages

No □, Please complete below and the following day

Number of stools a day ____________________________(numbers)

Have you had:	No	Yes	Further comments
Watery stools	□	□	______________________
Mucus in stools	□	□	______________________
Blood in stools	□	□	______________________
Noctural diarrhoea	□	□	______________________
Fever	□	□	Temperatur_______________C°
Chills	□	□	______________________
Nausea	□	□	______________________
Vomiting	□	□	Numbers________________________
Headache	□	□	______________________
Dizziness	□	□	______________________
Abdominal pain	□	□	______________________
Muscle aches	□	□	______________________
Weight loss	□	□	If yes, write weight _______kg

Other symptoms

____

Side effects □ Yes □ No □ Please explain:

____

____

Questionnaire Day 8 Date (ddmmyy) ____________________

Are you cured: Yes □, You do not have to fill the next pages

No □, Please complete below and the following day

Number of stools a day ____________________________(numbers)

Have you had:	No	Yes	Further comments
Watery stools	☐	☐	______________________________
Mucus in stools	☐	☐	______________________________
Blood in stools	☐	☐	______________________________
Noctural diarrhoea	☐	☐	______________________________
Fever	☐	☐	Temperatur_______________C°
Chills	☐	☐	______________________________
Nausea	☐	☐	______________________________
Vomiting	☐	☐	Numbers_______________________
Headache	☐	☐	______________________________
Dizziness	☐	☐	______________________________
Abdominal pain	☐	☐	______________________________
Muscle aches	☐	☐	______________________________
Weight loss	☐	☐	If yes, write weight _______kg

Other symptoms
____

Side effects ☐ Yes ☐ No ☐ Please explain:
____
____

Questionnaire Day 9 Date (ddmmyy) ____________________

Are you cured: Yes □, You do not have to fill the next pages

No □, Please complete below and the following day

Number of stools a day ____________________________(numbers)

Have you had:	No	Yes	Further comments
Watery stools	☐	☐	________________
Mucus in stools	☐	☐	________________
Blood in stools	☐	☐	________________
Noctural diarrhoea	☐	☐	________________
Fever	☐	☐	Temperatur_______________C°
Chills	☐	☐	________________
Nausea	☐	☐	________________
Vomiting	☐	☐	Numbers________________
Headache	☐	☐	________________
Dizziness	☐	☐	________________
Abdominal pain	☐	☐	________________
Muscle aches	☐	☐	________________
Weight loss	☐	☐	If yes, write weight _______kg

Other symptoms

____

Side effects ☐ Yes ☐ No ☐ Please explain:

____

____

Questionnaire Day 10 Date (ddmmyy) ____________________

Are you cured: Yes ☐, You do not have to fill the next pages

No ☐, Please complete below and the following day

Number of stools a day ____________________________(numbers)

Have you had:	No	Yes	Further comments
Watery stools	☐	☐	______________________________
Mucus in stools	☐	☐	______________________________
Blood in stools	☐	☐	______________________________
Noctural diarrhoea	☐	☐	______________________________
Fever	☐	☐	Temperatur_______________C°
Chills	☐	☐	______________________________
Nausea	☐	☐	______________________________
Vomiting	☐	☐	Numbers_______________________
Headache	☐	☐	______________________________
Dizziness	☐	☐	______________________________
Abdominal pain	☐	☐	______________________________
Muscle aches	☐	☐	______________________________
Weight loss	☐	☐	If yes, write weight _______kg

Other symptoms
____

Side effects ☐ Yes ☐ No ☐ Please explain:
____
____
